# A blunted T_H_17 cytokine signature in women with mild cognitive impairment: insights from inflammatory profiling of a community-based cohort of older adults

**DOI:** 10.1093/braincomms/fcad259

**Published:** 2023-10-07

**Authors:** Adam D Bachstetter, Jenny Lutshumba, Edric Winford, Erin L Abner, Barbra J Martin, Jordan P Harp, Linda J Van Eldik, Frederick A Schmitt, Donna M Wilcock, Ann M Stowe, Gregory A Jicha, Barbara S Nikolajczyk

**Affiliations:** Spinal Cord and Brain Injury Research Center, University of Kentucky, Lexington, KY 40536, USA; Department of Neuroscience, University of Kentucky, Lexington, KY 40536, USA; Sanders-Brown Center on Aging, University of Kentucky, Lexington, KY 40536, USA; Spinal Cord and Brain Injury Research Center, University of Kentucky, Lexington, KY 40536, USA; Sanders-Brown Center on Aging, University of Kentucky, Lexington, KY 40536, USA; Department of Neuroscience, University of Kentucky, Lexington, KY 40536, USA; Sanders-Brown Center on Aging, University of Kentucky, Lexington, KY 40536, USA; Department of Epidemiology, University of Kentucky, Lexington, KY 40536, USA; Sanders-Brown Center on Aging, University of Kentucky, Lexington, KY 40536, USA; Sanders-Brown Center on Aging, University of Kentucky, Lexington, KY 40536, USA; Department of Neurology, University of Kentucky, Lexington, KY 40536, USA; Department of Neuroscience, University of Kentucky, Lexington, KY 40536, USA; Sanders-Brown Center on Aging, University of Kentucky, Lexington, KY 40536, USA; Spinal Cord and Brain Injury Research Center, University of Kentucky, Lexington, KY 40536, USA; Sanders-Brown Center on Aging, University of Kentucky, Lexington, KY 40536, USA; Department of Neurology, University of Kentucky, Lexington, KY 40536, USA; Department of Behavioral Sciences, University of Kentucky, Lexington, KY 40536, USA; Department of Neuroscience, University of Kentucky, Lexington, KY 40536, USA; Sanders-Brown Center on Aging, University of Kentucky, Lexington, KY 40536, USA; Department of Physiology, University of Kentucky, Lexington, KY 40536, USA; Department of Neuroscience, University of Kentucky, Lexington, KY 40536, USA; Department of Neurology, University of Kentucky, Lexington, KY 40536, USA; Sanders-Brown Center on Aging, University of Kentucky, Lexington, KY 40536, USA; Department of Neurology, University of Kentucky, Lexington, KY 40536, USA; Department of Pharmacology and Nutritional Science, and Barnstable Brown Diabetes and Obesity Center, University of Kentucky, Lexington, KY 40536, USA

**Keywords:** Alzheimer’s disease, neuroimmunology, biomarker, immunity, sex differences

## Abstract

People with dementia have an increase in brain inflammation, caused in part by innate and adaptive immune cells. However, it remains unknown whether dementia-associated diseases alter neuro-immune reflex arcs to impact the systemic immune system. We examined peripheral immune cells from a community-based cohort of older adults to test if systemic inflammatory cytokine signatures associated with early stages of cognitive impairment. Human peripheral blood mononuclear cells were cultured with monocyte or T-cell-targeted stimuli, and multiplex assays quantitated cytokines in the conditioned media. Following T-cell-targeted stimulation, cells from women with cognitive impairment produced lower amounts of T_H_17 cytokines compared with cells from cognitively healthy women, while myeloid-targeted stimuli elicited similar amounts of cytokines from cells of both groups. This T_H_17 signature correlated with the proportion of circulating CD4+ and CD8+ T cells and plasma glial fibrillary acidic protein and neurofilament light concentrations. These results suggest that decreases in T_H_17 cytokines could be an early systemic change in women at risk for developing dementia. Amelioration of T_H_17s cytokines in early cognitive impairment could, in part, explain the compromised ability of older adults to respond to vaccines or defend against infection.

## Teaser

Bachstetter *et al*. found diminished T_H_17 cytokines produced by activated T cells from women with early cognitive decline, indicating a dementia-linked systemic immunological shift associated with the proportion of circulating CD4+ and CD8+ T cells and plasma glial fibrillary acidic protein and neurofilament light concentrations.

## Graphical Abstract

**Graphical Abstract:**
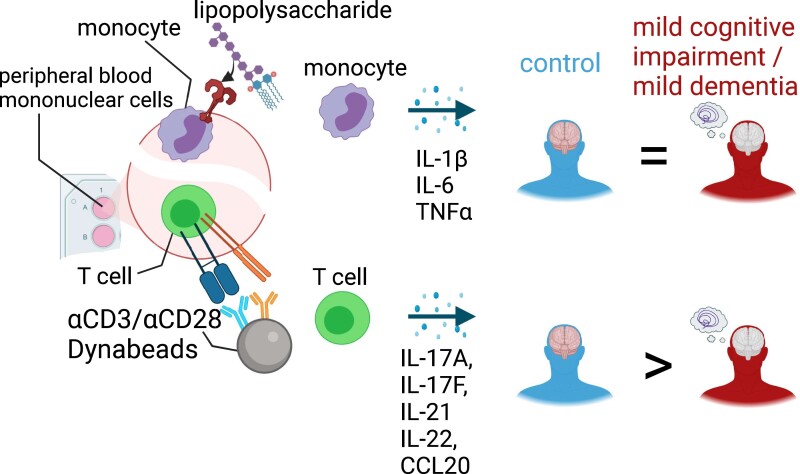

## Introduction

Neuroinflammation is a prominent feature in the brains of people who die with dementia.^1,2^ Moreover, genetics strongly implicate immune system involvement in the pathogenesis of Alzheimer’s disease neuropathologic changes (ADNCs) that result in dementia.^3,4^ The mapping of the immune system landscape in the brains of people with neurodegenerative disease has moved rapidly over the last two decades, providing exciting ways to target disease progression.^5^ However, while much is known about brain tissue immune responses, a similar understanding of the systemic immune system in ADNC is lacking.

Compared with other tissues, the CNS witnesses limited immune cell exchange, but does have active immune surveillance^6^ as part of the multiple bidirectional communication paths that have remarkable bidirectional physiological effects on both immune and nervous systems.^6,7^ Both delirium and sickness behaviour illustrate the connection of the immune system to the nervous system, as a systemic infection may cause either phenomenon without detectable CNS infection.^8,9^ Other examples of the nervous-to-immune system connection include CNS injury-induced immunodeficiency (also called immunosuppression or immunodepression) that occurs after stroke, or traumatic brain, or spinal cord injury.^10,11^

Older individuals with dementia have high rates of infections (e.g. pneumonia, urinary tract infection, COVID-19), which can be a primary cause of hospitalization, delirium, and death.^12,13^ Yet only a handful of studies have evaluated the systemic adaptive immune system in AD or AD-related animal models.^14,15^ The results of these studies highlight the complexity by which the immune system can influence the brain. For instance, blockade of adaptive immune cell checkpoints, such as the PD-1, improves outcomes in multiple AD-relevant animal models.^16-18^ Similarly, the lack of an adaptive immune system worsens pathology and neuroinflammation in an AD-relevant mouse model, potentially through interactions with the tissue-resident immune cells, microglia.^19^ In people, the number of systemic adaptive immune cells in the blood decreases with age, while the number of innate immune cells expands. Dementia further tilts this balance in favour of innate immunity.^14^ Although, CD4+ and CD8+ T cells have been associated with dementia with Lewy bodies (DLB) and AD-type dementia, respectively,^20,21^ the physiology of the systemic immune cells in early-stage cognitive impairment is unknown.

We recruited a community-based cohort of older individuals to test the hypothesis that cytokine production by systemic immune cells in response to cognate stimuli identifies an inflammatory signature that associates with early stages of cognitive impairment. We report here lower production of T_H_17 family cytokines in women, but not men, with cognitive impairment.

## Materials and methods

### Human subjects

Study participants were selected from a larger University of Kentucky Alzheimer Disease Research Center (UK-ADRC) community-based cohort study of ageing and dementia. Informed consent was obtained following University of Kentucky Institutional Review Board-approved protocols. As previously described,^22^ the UK-ADRC study recruits older adult participants from the central Kentucky region of the USA, and includes ∼500 active participants that have annual follow-up visits. These visits include physical examination, neurological examination, medical history, and cognitive testing, among other measures. Clinical evaluations and cognitive testing were completed as previously described and followed the National Alzheimer’s Coordinating Center uniform data set used by Alzheimer’s disease centres.^23,24^ From the UK-ADRC cohort, we assayed peripheral blood from *n* = 44 cognitively healthy [Clinical Dementia Rating score (CDR) = 0], and *n* = 40 cognitively impaired (CDR = 0.5–1) individuals, with groups statistically similar for gender, and cardiovascular disease (CVD) risk factors (hypertension, hyperlipidaemia, or obesity), and age as indicated by their previous year study visit data. Exclusion criteria included Type 2 diabetes, autoimmune disease, and current cancers. Blood samples were collected from August 2020 until June 2021. The study coordinator (B.J.M.) provided the de-identified samples to the experimentalists (A.D.B., J.L., and B.S.N.), who were blinded to the participant group identity until all the data had been generated.

### Peripheral blood mononuclear cell isolation

Five to 10 mL of peripheral blood was collected into acid/citrate/dextrose-containing tubes by venous puncture. The peripheral blood mononuclear cells (PBMCs) were purified by density centrifugation in Ficoll histopaque 1077 using SepMate PBMC isolation tube (StemCell Technologies, Cat#: 85415) according to the manufacturer’s protocol. The cells were then washed in buffer [0.1 BSA, 2 mM EDTA (ethylenediaminetetraacetic acid), 1× PBS]. Residual red blood cells were removed using red blood cells lysis buffer (eBioScience, Cat#: 00-4300-54), and the resulting PBMCs were aliquoted at a density of 1 × 10^6^/mL, in freezing media containing 90% heat-inactivated FBS, plus 10% DMSO. The cells were then frozen at −80°C in a Mr Frosty apparatus (Nalgene) for 24 h, before storing the cells in liquid N_2_, until use.

### Cell culture

After thawing rapidly in a 37°C water bath, the PBMCs were plated at a density of 10^6^ cells/mL/well in a 12-well plate (VWR, Cat#: 10062-894), in glucose-free RPMI media, containing L-glutamine and 25 mM hepes (Gibco, Cat#: 11879020) supplemented with 10% FBS heat-inactivated (Gibco, Cat#: 10438026), 1% pen/strep (Gibco, Cat#: 15140122), and 1% sodium pyruvate (Corning, Cat#: 25-000-CI). The cells were stimulated in multiple batches, and each batch included a random sampling of approximately six participants. The PBMCs were stimulated with *Escherichia coli* O111:B4 lipopolysaccharides (LPS) (25 ng/mL, Millipore Sigma; 20 h) or αCD3/αCD28 Dynabeads (1 bead/cell; Gibco, Cat#: 11131D; 40 h) in a CO_2_ incubator at 37°C prior to conditioned media collection. The unstimulated cells were incubated 40 h prior to conditioned media collection. The conditioned media was aliquoted and stored at −80°C for cytokine assays. The 20 and 40 h post-stimulation timepoints were determined based on prior studies.^25-28^

### Multiplex measurement of cytokine concentrations from stimulated PBMCs

Supernatant samples were thawed then centrifuged for 10 min at 1200 rpm to remove debris before being added to a 384-well plate for analysis by bioplex using the Milliplex human T_H_17 25-plex kit (Millipore) or 96-well Meso Scale Discovery (MSD) proinflammatory or T_H_17 multiplex kit, following the manufacturer’s protocols (Supplemental Tables 1 and 2). Samples were diluted in an assay buffer to ensure that the cytokines were in the linear range of the standard curve. The unstimulated cells were run undiluted. The MSD assay was used for cytokines that outside the linear range for the Milliplex kits.

### Plasma biomarker analysis

Plasma collected from the same blood draw providing PBMCs was analysed on the Quanterix Simoa HD-X as previously described^29^ or on the MSD Quickplex SQ 120 using the V-Plex T_H_17 panel 1 (MSD, Cat#: K15085D), following the manufacturer’s instructions.

### Flow cytometry

PBMCs were unthawed and washed with warm RPMI then washed with PBS. Cells were then stained with live/dead marker Ghost Dye-Alexa Fluor 700 (Tonbo Biosciences) for 30 min at 4°C according to the manufacturer’s instructions, then washed with FACS buffer. Human FcR blocking reagent, human (Miltenyi Biotec) was used to block unwanted binding of antibodies to FC receptors for 10 min at 4°C. Cells were then stained for immune cell markers CD3-FITC (BD Biosciences, Cat#: 555332), CD8-PE (BD Biosciences, Cat#: 555635), CD4-APC (BD Biosciences, Cat#: 555349) for 30 min at 4°C. Stained cells were fixed with cytofix (BD Biosciences) for 30 min at 4°C, then washed and suspended in FACS buffer (PBS with 1% BSA and 0.1% sodium azide). BD Symphony A3 was used to acquire cell populations. Analysis was conducted with Flow v10.8 software. Fluorescence-minus one controls were used to identify cell populations, and each experiment had single-stained and no-stain controls.

### Statistics

Characteristics of participants were assessed with ANOVA, or *χ*^2^ tests. All cytokine and fluid biomarker data were log_2_ transformed. For categorical comparisons, an unadjusted *t*-test or one-way ANOVA were used for the combined data, or for data stratified by sex. Multiple linear regression models were used to adjust mean responses for covariates as listed in the figure and Supplemental tables. False discovery rate (FDR) values for multiple comparisons were applied based on the Benjamini–Hochberg adjustment. Pairwise effect sizes were calculated using Cohen’s *d*. The effect size for linear regressions was calculated as *R*^2^ or Ω^2^. *Z*-scores were created using the sample means and standard deviations of the log_2_ transformed data. For the box-and-whiskers plots, the box shows the median and 25th and 75th percentile, and the whiskers show the minimum and maximum values. All data analysis was completed in JMP Pro v16 between January 2022 and September 2022. Data visualizations were created in JMP Pro v16 or GraphPad Prism v9.4.

## Results

Participants for the study (*N* = 84) were selected from the larger community-based cohort study of ageing and dementia conducted by the UK-ADRC. Table 1 shows the demographic characteristics of the study participants. Study recruitment was designed to balance gender, cognitive status (CDR = 0 versus CDR = 0.5–1), age, and CVD risk factors (hypertension, hyperlipidaemia, or obesity). There were no statistically significant differences between or within the groups formed by gender and cognitive status.

**Table 1:** Participant characteristics

	Women		Men	
	CDR = 0	CDR = 0.5–1	CDR = 0	CDR = 0.5–1
*N*	24	20	16	24
Age years (mean ± SD)	76 ± 7.9	79 ± 7.4	79 ± 7.6	80 ± 8.2
APOE E4^a^ allele (*n*)	6	12	3	8
Hypertension [*n* (%)]	13 (54.2%)	12 (60%)	11 (68.8%)	13 (54.2%)
Hyperlipidaemia [*n* (%)]	14 (56%)	13 (65%)	11 (68.8%)	14 (58.3%)
BMI (mean ± SD)	26.3 ± 3.75	25.3 ± 4.6	26.6 ± 3.3	26.5 ± 3.7
Education years (mean ± SD)	16.5 ± 2.6	15.7 ± 2.6	17.9 ± 2.3	17.5 ± 2.9
MMSE (mean ± SD)	29.0 ± 1.4	22.8 ± 6.6	29.2 ± 1.0	24.8 ± 5.5
MoCA (mean ± SD)	26.5 ± 2.5	17.2 ± 5.9	27.1 ± 1.6	19.9 ± 6.2

BMI, body mass index; CDR, Clinical Dementia Rating scale; MMSE, Mini-Mental State Examination; MoCA, Montreal Cognitive Assessment.

^a^One or two copies.

### Association between CDR and systemic innate immune cell activation

**Figure 1:**
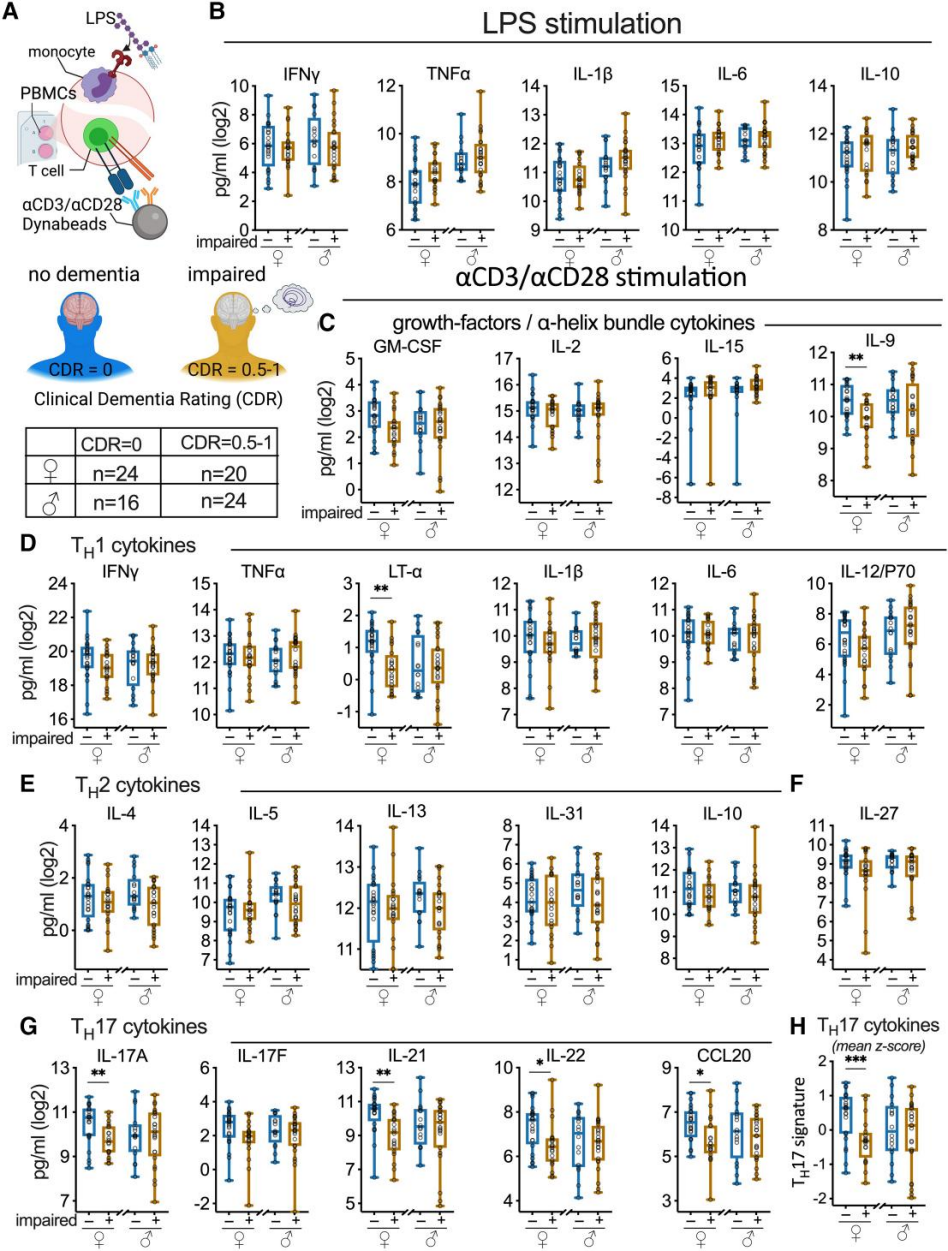
**T_H_17 cytokines from αCD3/αCD28-stimulated PBMC associate with cognitive impairment in women.** (**A**) PBMCs were stimulated with LPS or αCD3/αCD28. Study participants were divided into healthy controls (CDR = 0) or cognitively impaired (CDR = 0.5–1). (**B**) Cognitive status had no effect on levels of cytokines produced by the LPS-stimulated PBMCs. The cytokines produced by αCD3/αCD28-stimulated PBMCs were clustered into functional categories, including (**C**) cytokines that promote growth and survival of T cells; (**D**) T_H_1-type cytokines that promote cellular immune and inflammatory responses to infection and injury, (**E**) T_H_2-type cytokines associated with allergy and anti-inflammatory activity; (**F**) IL-27 associated with polarization of T cells from T_H_1/T_H_2 to a T_H_17 response; and (**G**) T_H_17 cytokines associated with activating cellular immunity and autoimmune disease. (**H**) The T_H_17 cytokines (IL-17A, IL-17F, IL-21, IL-22, CCL20) were standardized and the average *z*-score was used to identify a T_H_17 signature. Cognitively impaired women had a lower T_H_17 signature than control women. Circles represent individual study participants. *FDR < 0.05, **FDR < 0.01, ***FDR < 0.001 for unadjusted *t*-test. See Supplemental Tables 4 and 5 for all statistical comparisons. Multiple comparisons corrected with the Benjamini–Hochberg FDR.

ADNC-associated genetic polymorphisms implicate innate immune cell function in dementia.^3^ Therefore, we hypothesized that PBMCs from research participants with cognitive impairment (CDR = 0.5–1) would produce more cytokines following LPS stimulation (25 ng/mL *E. coli*, for 20 h) (Fig. 1A), which predominantly targets innate immune cells, compared with cells from participants with normal cognition (CDR = 0). Because sex-dependent differences in inflammation and dementia are documented,^30-32^ the data were stratified to evaluate PBMCs from women and men independently. We found degree of cognitive impairment based on CDR ratings did not associate with cytokine production by unstimulated or LPS-stimulated PBMCs (Fig. 1B; Supplemental Fig. 1). LPS responses by cells from unimpaired and impaired individuals remained similar after adjustment for age and CVD risk (Supplemental Tables 4 and 5).

### Alterations in T-cell function associated with cognitive impairment

Alterations in CD4^+^ and CD8^+^ T-cell populations have been reported in patients with AD and DLB,^20,21^ consistent with studies from the 1980s that found T cells from patients with probable AD to be less proliferative in response to stimulation than T cells from healthy controls (for review^14^). Based on this prior work, we hypothesized that cells from participants with cognitive impairment would show a different profile of T-cell-associated cytokine production compared with cells from cognitively normal subjects. To evaluate the association of cognitive impairment with T-cell function, we grouped the 22 cytokines induced by αCD3/αCD28 Dynabeads stimulation (1 bead/cell for a 40 h) into functional categories for growth factors, T_H_1, T_H_2, and T_H_17 cytokines (Fig. 1C–H; Supplemental Tables 4 and 5).

PBMCs from women with cognitive impairment produced lower concentrations of cytokines following T-cell stimulation compared with PBMCs from women without cognitive impairment (Fig. 1C–H; Supplemental Table 4). IL-9, a cytokines associated with proliferation and survival of T cells (Fig. 1C; Supplemental Table 4), was lower in women with cognitive impairment. In addition, lymphotoxin alpha (LTα), which has an important function in stimulating cellular immunity, was lower in women with cognitive impairment (Fig. 1D; Supplemental Table 4). Strikingly, the family of cytokines associated with a T_H_17 response was produced at lower concentrations by PBMCs from women with cognitive impairment compared with those without cognitive impairment (Fig. 1G; Supplemental Table 4). We transformed five T_H_17-related cytokines (IL-17A, IL-17F, IL-21, IL-22, CCL20) into a single variable by standardizing the cytokines based on sample means and standard deviations to create a mean *Z*-score value that we define as a T_H_17-associated cytokine signature. This T_H_17 cytokine signature was produced in lower concentrations by PBMCs from impaired compared with unimpaired women (Fig. 1H; Supplemental Table 6), in agreement with the individual cytokine measurements (Fig. 1G). Our statistical models lacked strong evidence for an interaction of age (Model 1), or age and CVD risk (Model 2) with T_H_17 signature (Supplemental Table 6). In sharp contrast, following αCD3/αCD28 stimulated PBMCs from men produced cytokines that were largely independent of cognitive status; this lack of association remained after accounting for covariates (Fig. 1C–H; Supplemental Table 5).

### Association of covariates with cytokine response from stimulated PBMCs

**Figure 2:**
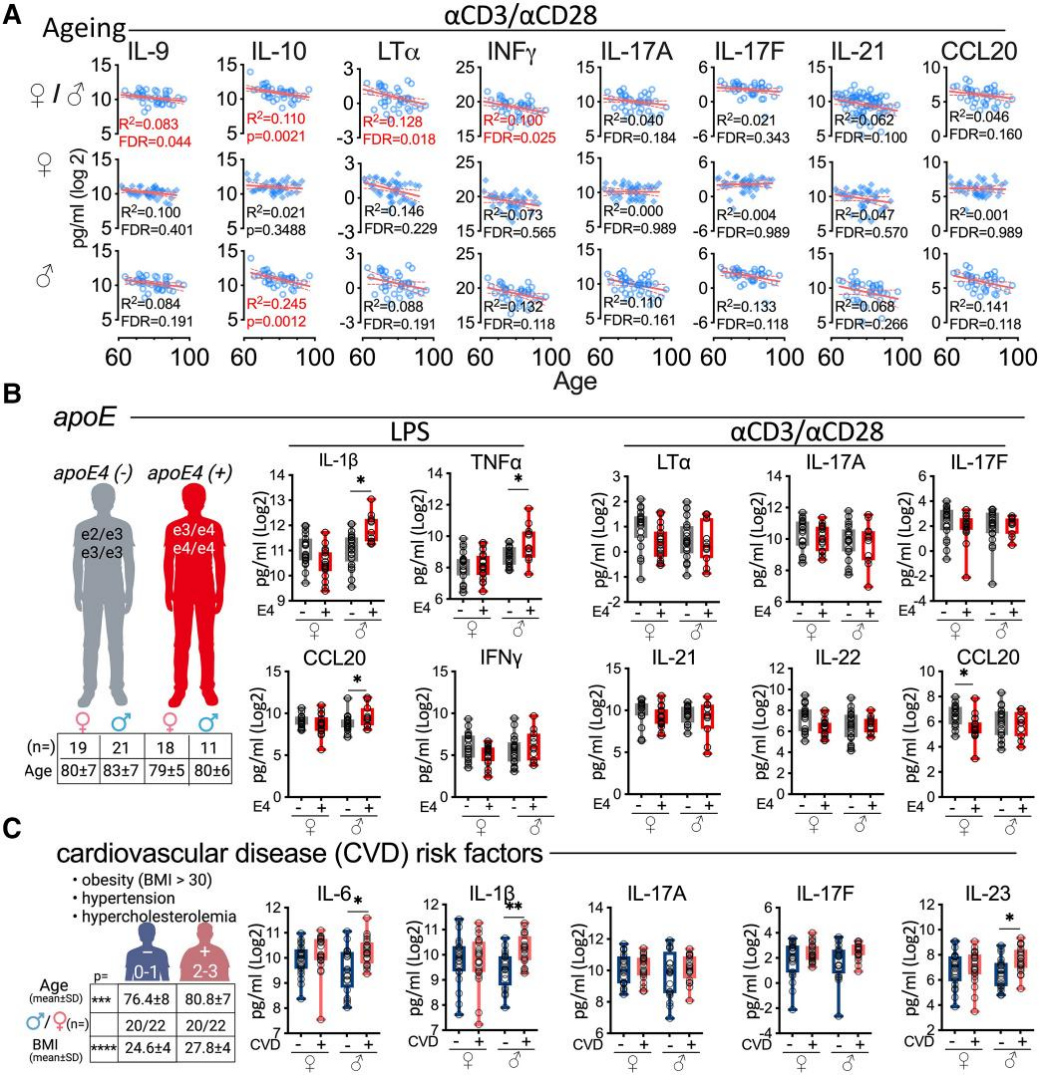
**The association of age, *apoE* genotype, and CVD risk factors with cytokine production from stimulated PBMCs.** (**A**) Increased age correlated with lower amounts of cytokines produced from αCD3/αCD28-stimulated PBMCs. The correlation was gender-dependent, particularly with the T_H_17-associated cytokines, where age correlated with lower cytokines in men but not women. (**B**) In men, *ApoE4* carriers (one or two copies) had higher IL-1β, TNFα, and CCL20 from LPS-stimulated PBMCs compared with the *E4*− group. In women, stimulated *E4*+ PBMCs produced fewer cytokines (IFNγ, LTα, IL-22, and CCL20) than stimulated *E4*− PBMCs. Age was not different between the *E4*− or *E4*+ groups (*t* = −1.01, *P* = 0.32). (**C**) In men, a greater burden of CVD risk factors (2–3 versus 0–1) was associated with more cytokines produced from αCD3/αCD28-stimulated PBMCs. Women did not show the same pattern. Circles are individual participants. The dashed line shows the 95% confidence interval for the linear regression (**A**). *FDR < 0.05, **FDR < 0.01, ***FDR < 0.001, unadjusted *t*-test results (**B** and **C**). See Supplemental Tables 7–10 for all statistical comparisons. The Benjamini–Hochberg adjustment used for FDR values.

We next evaluated putative inflammatory covariates of age, *apoE* genotype, and CVD risk factors, independent of cognitive status, to better understand their possible contribution to the cytokine response from the stimulated PBMCs. Age did not associate with cytokine response elicited from PBMCs by LPS stimulated PBMCs, including for IL-1β and TNFα production (Supplemental Fig. 2; Supplemental Table 7). In contrast, four cytokines (IL-9, IL-10, LTα, and IFN-γ) elicited by αCD3/αCD28 stimulation negatively correlated with age (Fig. 2A; Supplemental Table 7). PBMCs from men (but not women) showed a modest-to-strong effect size (*R*^2^ = 0.11–0.14) for a lower IL-17A, IL-17F, and CCL20 response with age following αCD3/αCD28 stimulation (Fig. 2A; Supplemental Table 7). We conclude age is not a major variable driving the lower T_H_17-associated cytokines from αCD3/αCD28-stimulated PBMCs in women with cognitive impairment, but age may impact outcomes in cells from men.

*ApoE* genotype is known to influence inflammatory responses,^33-35^ but the effect of ApoE genotype on systemic immune responses is less understood. Of the 69 individuals whose ApoE genotype was provided, 29 individuals (34.5%) had 1 or 2 copies of *apoE4* (Fig. 2B). LPS-stimulated PBMCs from male *E4* carriers produced more IL-1β, TNFα, and CCL20 than PBMCs from *E4* null men (Fig. 2B; Supplemental Table 8). αCD3/αCD28-stimulated PBMCs from *E4* women made less CCL20 compared with cells from *E4* null women (Fig. 2B; Supplemental Table 8). We found no interaction between three other T_H_17-associated cytokines (IL-17A, IL-17F, IL-21) and the *ApoE* genotype. These results support the interpretation that the T_H_17-associated cytokine changes seen in women with cognitive impairment cannot be explained by *ApoE* genotype.

CVD risk factors are known to modulate inflammation^36^ thus we tested the possibility that CVD risk factors impact associations between cytokine production and cognitive status. LPS-elicited cytokine production was unaffected by CVD risk factor status (Fig. 2C; Supplemental Table 9). In contrast, the αCD3/αCD28-stimulated PBMCs from high CVD-risk men (2–3, versus 0–1) produced more cytokines, while there was no effect of CVD status on cytokine production by cells from women (Fig. 2C; Supplemental Table 9). Despite our best attempts, younger age and body mass index (BMI) of the low CVD risk group (*P* = 0.008 and *P* < 0.0001, respectively) putatively impact these associations.

Follow-up work evaluated each CVD risk factors independently. αCD3/αCD28-stimulated cells from men with hypertension produced higher concentrations of IL-1β, likely as an indirect response of myeloid cells to primary T-cell responses compared with cells from men without hypertension (Supplemental Fig. 3; Supplemental Table 10). Furthermore, αCD3/αCD28-stimulated PBMCs from men with hypercholesterolaemia produced more of IL-1β, and IL-6 compared with cells from normolipidaemic men (Supplemental Fig. 4; Supplemental Table 10). There was no association between cytokine amounts and hypercholesterolaemia in women, nor between cytokine profiles and BMI of both sexes (Supplemental Table 11). These results demonstrate that hypertension and hypercholesterolaemia, make unique contributions to the pattern of cytokines changes associated with CVD risk factor status, contributing mainly to IL-1β and TNFα production.

CVD risk factors (Fig. 2) and cognitive impairment (CDR; Fig. 1) associated with alterations in the T_H_17 family of cytokines; therefore, we wanted to ensure that CVD risk was not a confounding variable in the T_H_17 cytokine signature associated with cognitive impairment. CVD risk did not influence the T_H_17 signature associated with cognitive impairment in women (Supplemental Table 9). Also, a two-way ANOVA found no interaction between CDR and CVD risk (*F* = 1.144, *P* = 0.288). We also found no evidence that the number of participants with CVD risk factors was unbalanced (Supplemental Fig. 5). Finally, stratifying the study participants by the CDR and CVD risk status, the strongest association with the T_H_17 cytokine signature in women was with the CDR group. Cognitively impaired women with either low or high CVD risk had a blunted T_H_17 signature compared with the cognitively unimpaired women (Supplemental Fig. 5). These results suggest that the T_H_17 cytokine signature is not associated with CVD/cerebrovascular-related cognitive decline.

Finally, we evaluated the supplements and medications taken by each participant, including statins, antihistamines, and antidepressants [few participants reported non-steroidal anti-inflammatory drug (NSAID) use]. While we cannot entirely rule out medication effects, we did not find medication usage that coincides with the T_H_17 cytokine signature.

### Association of T_H_17 cytokine signature with mild cognitive impairment

**Figure 3:**
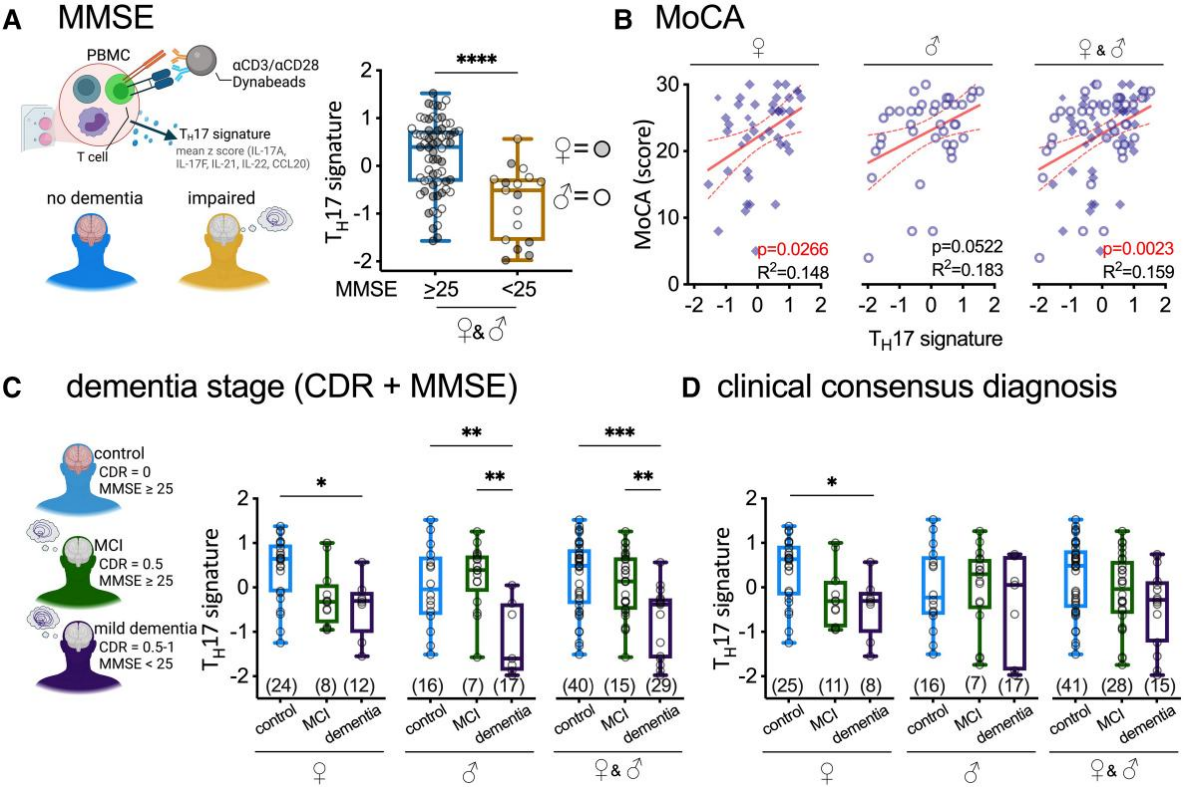
**PBMCs from cognitively impaired subjects make lower amounts of T_H_17-associated cytokines than cognitively unimpaired subjects.** (**A**) Individuals with cognitive impairment on the MMSE (score <25) had a low T_H_17 signature (mean *Z*-score of IL-17A, IL-17F, IL-21, IL-22, CCL20) compared with those within a normal MMSE range (score ≥25) (****P < 0.0001; corrected for age and education). (**B**) The MoCA correlated with the T_H_17 signature (age and education adjusted). The dashed line shows the 95% confidence interval for the linear regression. (**C**) Staging for dementia was based on CDR and MMSE results. The T_H_17 signature was lower in women with MCI and mild dementia. In men, the lower T_H_17 signature was only seen in individuals with mild dementia. When including both genders, the decline in the T_H_17 signature was only seen with mild dementia and not MCI (*P < 0.05, **P < 0.01, ***P < 0.001, Tukey test). (**D**) The neuropsychological consensus diagnosis for the visit when PBMCs were collected also showed a significant association of a lower T_H_17 signature with dementia (*P < 0.05, Tukey test). Markers are individual participants. Statistical tests used: (**A**) *t*-test, (**B**) linear regression, (**C** and **D**) one-way ANOVA with Tukey *post hoc* test.

We used additional global cognitive tests with increased granularity and sensitivity to detect early cognitive function changes to determine if the lower T_H_17 cytokine signature was present in study participants with mild cognitive impairment (MCI). The first alternative measure of global cognition we tested was the Mini-Mental State Examination (MMSE) (Fig. 3A), which was administered independently from the CDR. Most of the study participants (80%) had an MMSE score of >25; therefore, rather than linear regression, we used a standard cut-point of <25 on the MMSE to identify individuals with cognitive impairment. Although, the low number of impaired individuals on the MMSE test (*n* = 17) prevented data stratification by gender, the PBMCs from those individuals compared with subjects with MMSE < 25 produced significantly less T_H_17-associated cytokines (Fig. 3A) [*F*(3,81) = 19.14, *P* < 0.0001, Cohen’s *d* = 1.3, age + education adj.].

While the MMSE and the Montreal Cognitive Assessment (MoCA) are both scored on a 30-point scale and are widely reported measures of global cognitive function, the MoCA is a more challenging test that includes measures of executive function, language, and visuospatial processing. The MoCA is useful for distinguishing mild levels of cognitive impairment and has a higher discriminant potential than the MMSE for detecting MCI.^37^ Lower scores on the MoCA, which indicate cognitive impairment, strongly correlated with the T_H_17 cytokine signature (Fig. 3B) [*F*(3,81) = 9.95, *P* = 0.002, Ω^2^ = 0.099, age + education adj.]. After stratification by gender, both men and women showed a similar association between the T_H_17 cytokine signature and the MoCA. However, after adjusting for age and education, only in women did the correlation reached statistical significance (Fig. 3B) [*F*(3,40) = 5.33, *P* = 0.027, Ω^2^ = 0.099 age + education adj.].

The MoCA results suggest that changes in the T_H_17 cytokine signature may mark early stages of cognitive impairment. We tested this prediction using two standard approaches. First, we used a numeric approach by combining the clinician’s rating of cognitive complaints and functional impairments (CDR) and the MMSE score, following established methods to stratify the study participants with MCI from those with mild dementia.^32^ The MCI group was defined by CDR = 0.5, and an MMSE score ≥25. On the other hand, those with mild dementia were defined by a CDR = 0.5 and MMSE score <25, or CDR = 1. The gender-stratified data showed a unique pattern in the T_H_17 cytokine signature between women and men. In women, amelioration of the T_H_17 cytokine signature was detectable in the MCI phase and persisted into the mild dementia stage (Fig. 3C; Supplemental Table 12). In contrast, the T_H_17 cytokine signature in men declined only in the mild dementia stage (Fig. 3C; Supplemental Table 12).

For our second approach, we used the clinical consensus diagnosis for the visit when the PBMCs were collected (Fig. 3D). The consensus diagnosis considers multiple clinical and cognitive evaluations included in the National Alzheimer’s Coordinating Center Uniform data set and is determined by agreement between the UK-ADRC neurologists and neuropsychologists. In 80% of subjects, dementia stage (CDR + MMSE) aligned with the consensus diagnosis. For women, we again see a lower T_H_17 cytokine signature occurring in the MCI group, and also differed between the dementia group compared with the cognitively normal group. In contrast, we do not see an association between T_H_17 cytokine signature and consensus diagnosis in men.

### T_H_17 cytokine signature correlates with plasma biomarkers of neurodegeneration and neuroinflammation

**Figure 4:**
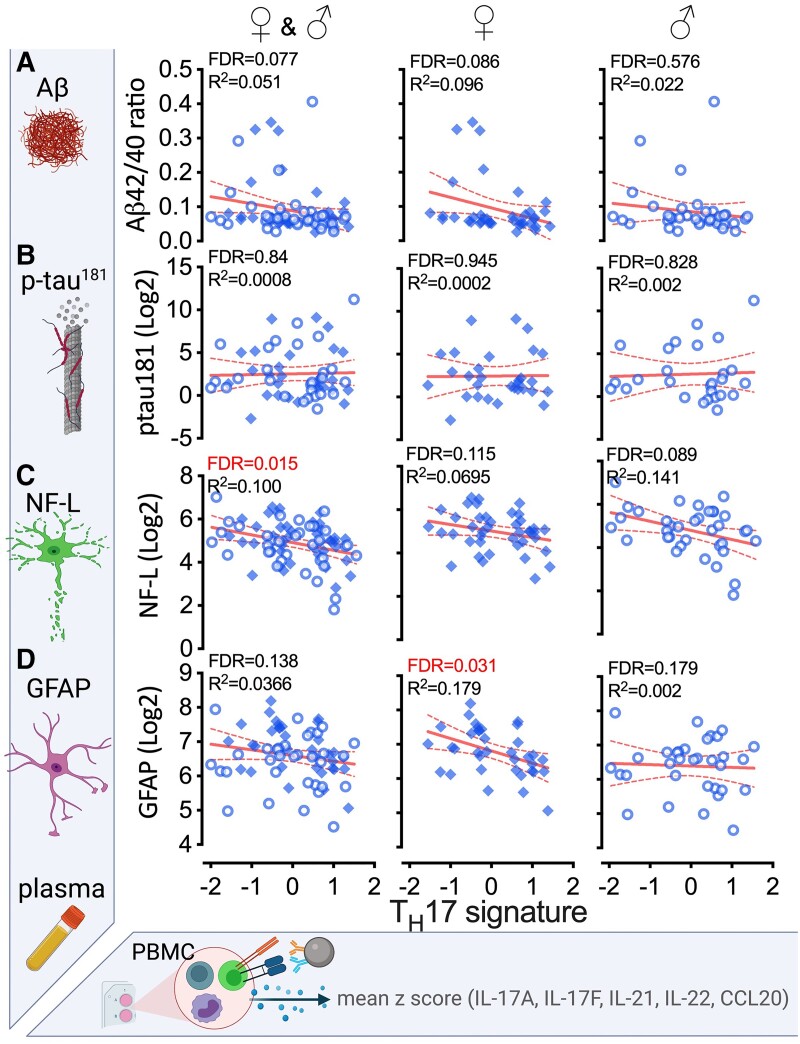
**The T_H_17 cytokine signature correlates with fluid biomarkers of neuronal injury and neuroinflammation**. Plasma biomarkers, measured by Simoa assays, were correlated with the T_H_17 cytokine signature (mean *z*-score of IL-17A, IL-17F, IL-21, IL-22, CCL20) of the αCD3/αCD28 Dynabead-stimulated PBMCs. Aβ42/40 ratio (**A**) and p-tau^181^ (**B**) levels in plasma did not correlate with the T_H_17 cytokine signature. NF-L (**C**) and GFAP (**D**) were negatively correlated with the T_H_17 cytokine signature. Open circles are male participants. Closed diamonds are female participants. The dashed line shows 95% confidence interval for the linear regression. FDR values are for the unadjusted analysis. See also Supplemental Table 13.

Comparing the pattern of changes between the MoCA, dementia staging, and consensus diagnosis suggested that the blunted T_H_17 cytokine signature may be associated with different domains of cognitive decline or sensitive to different aetiologies causing the cognitive decline. Therefore, we tested the hypothesis that the T_H_17 cytokine signature correlates with ADNC-related plasma biomarkers. Data for the Aβ42/Aβ40 ratio in plasma biomarkers were available for 75 (89%) participants. We did not find an association between the Aβ42/Aβ40 ratio and the T_H_17 cytokine signature (Fig. 4A). In contrast to the lack of association between the Th17 signature and Aβ42/Aβ40 ratio, the positive correlation between the T_H_17 cytokine signature and plasma Aβ40 was highly significant (FDR = 0.002; *R*^2^ = 0.166) (Supplemental Fig. 6 and Table 13). In addition, stratifying the data by gender, we found a positive correlation of the T_H_17 cytokine signature with plasma Aβ40 in women (FDR = 0.012; *R*^2^ = 0.236), but not men. We did not find an association with p-tau^181^ (Fig. 4B) in the 57 (68%) participants who had useable p-tau^181^ values. Lack of data on 32% of subjects weakens possible conclusions on associations between the T_H_17 cytokine signature and p-tau^181^.

Higher levels of neurofilament light chain (NF-L) and glial fibrillary acidic protein (GFAP) in the plasma are biomarkers of neurodegeneration and neuroinflammation, respectively. Plasma levels of these two biomarkers were available for 77 (92%; NF-L) and 69 (82%; GFAP) participants. We found a significant negative correlation between NF-L (Fig. 4C) and GFAP (Fig. 4D), the latter driven by a significance in women. These findings suggest that lower T_H_17 cytokine production, indicative of cognitive decline, may be a response to neuronal injury (NF-L) and neuroinflammation (GFAP).

When comparing the plasma biomarkers of neurodegeneration with the CDR, our analysis revealed an association between GFAP and CDR status in women. However, upon adjusting for age, this association disappeared (Supplemental Table 14). No other significant associations were found between CDR status and any of the other examined plasma neurodegeneration biomarkers (Supplemental Table 14).

### Plasma cytokine concentrations lack the sensitivity to detect T_H_17 signature associated with cognitive impairment

**Figure 5:**
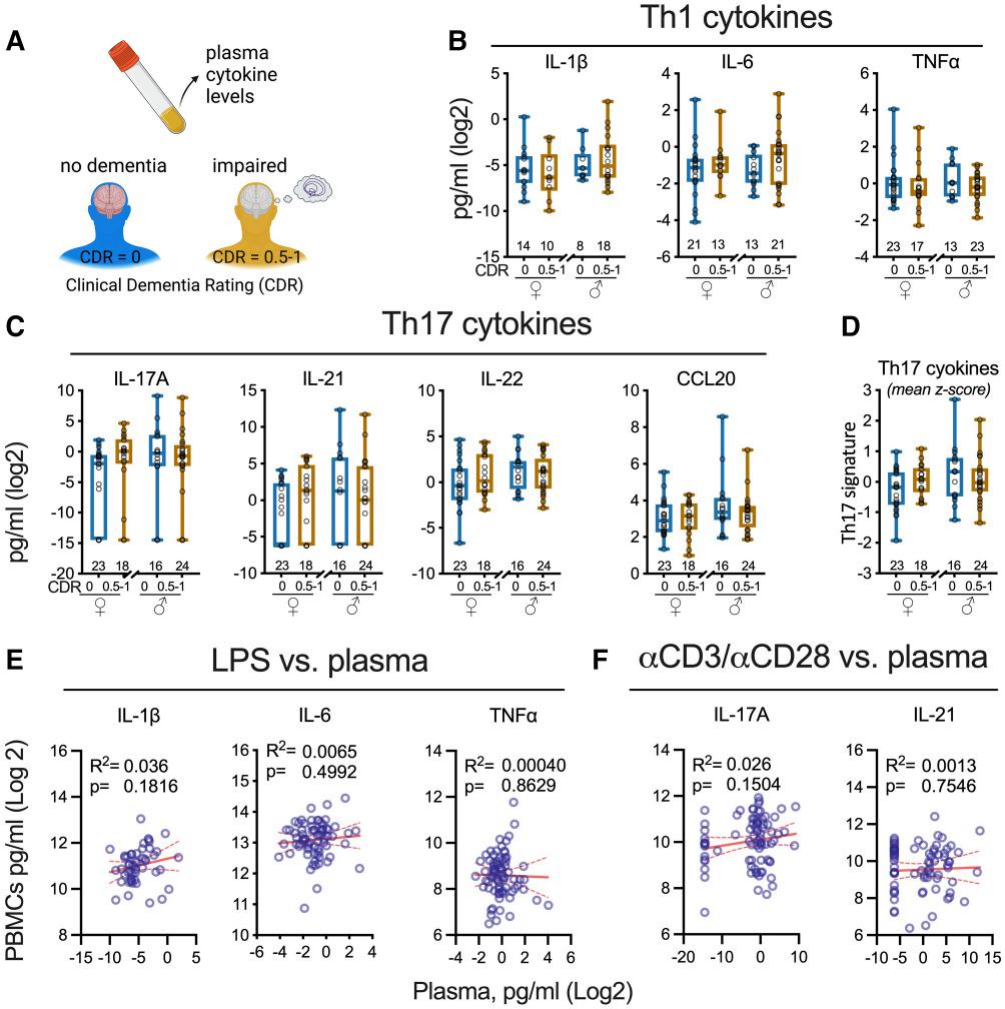
**Steady-state plasma cytokine levels fail to detect a dementia-associated immune signature.** (**A**) Cytokine levels were measured in plasma. There was no effect of cognitive impairment status on T_H_1 (**B**) or T_H_17 (**C**) cytokine levels in the plasma. A mean *z*-score (**D**) of the four T_H_17 cytokines measured in the plasma (**C**) did not define a dementia-associated immune signature. See also Supplemental Table 14. The plasma cytokine levels did not correlate with cytokines produced by LPS (**E**) or αCD3/αCD28 Dynabead (**F**) stimulated PBMCs. The dashed line shows 95% confidence interval for the linear regression. *P*-values are uncorrected. Markers are individual participants. FDR values are for the unadjusted analysis. See also Supplemental Table 15. Statistical tests used: (**A**–**D**) *t*-test, (**E** and **F**) linear regression. Multiple comparisons adjusted using the Benjamini–Hochberg FDR.

Prior studies have evaluated plasma cytokine levels in people with dementia and did not identify a T_H_17 signature, as seen with the αCD3/αCD28-stimulated PBMCs in this study.^38,39^ This raises at least two possibilities: (i) our study population is unique, or (ii) steady-state plasma cytokine levels do not correlate with cytokine production in *ex vivo* stimulated immune cells. To test these possibilities, we evaluated plasma concentrations of cytokines from the same blood draw used for PBMCs (Fig. 5A; Supplemental Table 15). Consistent with previous analyses, we found no association based between dementia status and commonly measured T_H_1 cytokine biomarkers (Fig. 5B), T_H_17 cytokines, (Fig. 5C), or the mean *z*-score for those cytokines (Fig. 5D). We found no association between steady-state plasma cytokine levels and cytokine production by stimulated PBMCs (Fig. 5E and F), consistent with cytokine outcomes in other inflammatory diseases we have studied.^25-28^ These data add to the growing appreciation that steady-state plasma cytokine levels lack the discriminative potential to detect disease-associated changes in circulating immune cell populations that could be actionable.

### The proportion of CD4^+^ T cells and the ratio of CD4^+^/CD8^+^ T cells is lower in women with cognitive impairment

**Figure 6:**
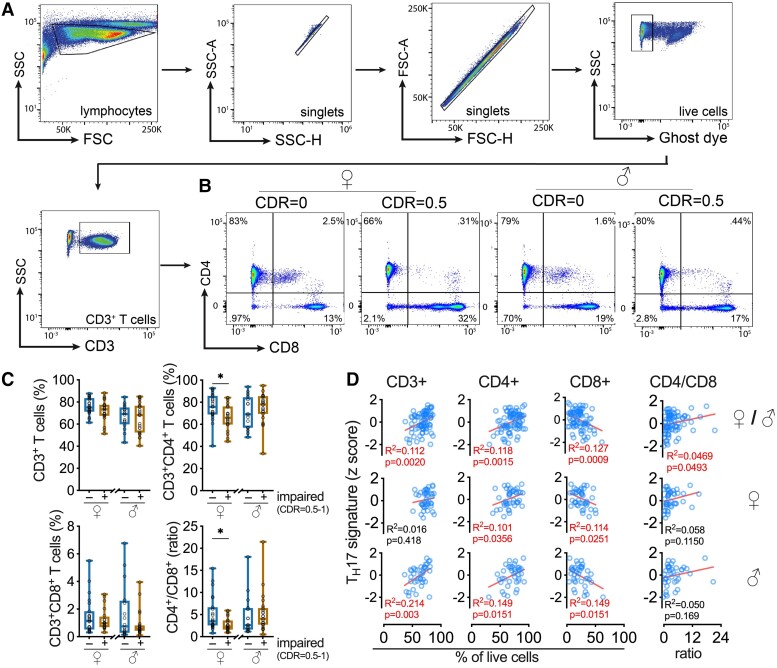
**The frequency of CD4+ T cells correlates with T_H_17 cytokine signature.** (**A**) Gating strategy and (**B**) representative example of CD4+ and CD8+ T-cell populations in the experimental groups. (**C**) A significant decrease in the percentage CD3+ CD4+ and CD4+/CD8+ ratio was found in women with cognitive decline compared with control women (FDR < 0.05, see also Supplemental Table 16). (**D**) The TH17 signature from stimulated PBMCs correlated with the percentage of T cells in the unstimulated PBMCs. Statistical tests used: (**C**) *t*-test and (**D**) linear regression.

We used flow cytometry to explore if alterations in CD4^+^ or CD8^+^ T-cell proportions associated with the TH17 signature. For these studies, it was imperative to analyse unstimulated cells, given αCD3/αCD28 causes CD4^+^, and to a lesser extent, CD8^+^ shedding among other surface marker changes. Our staining and gating strategies are shown in Fig. 6A and B. Women with cognitive impairment had similar frequencies of total (CD3^+^) or CD8^+^ T cells, but fewer CD4^+^ T cells, which explain the lower ratio of CD4^+^/CD8^+^ T cells compared with unimpaired women (Fig. 6C). These ratios were not affected by cognitive status in men (Fig. 6C). We detected a positive correlation between the composite T_H_17 signature and (i) CD3^+^ T cells; (ii) CD4^+^ T cells; and (iii) the CD4^+^/CD8^+^ T-cell ratio in analysis of all samples, but a negative correlation between the T_H_17 signature and CD8^+^ T cells (Fig. 6D, top row), although some of the relationship were insignificant in data analysed by gender (Fig. 6D, middle and bottom rows). These results suggest alterations in the pool of CD4^+^ T cells in the blood could contribute to the T_H_17 signature.

## Discussion

Here, we describe changes in systemic T cells that associated with cognitive impairment in women at the earliest stages of dementia, primarily lower production of T_H_17 cytokines by αCD3/αCD28-stimulated PBMCs, which we defined as a T_H_17 signature. While the paucity of CD4/T_H_17-related markers were notable at earlier stages of disease in women, T_H_17 cytokine production was also impaired in men with increasing severity of cognitive impairment. A decline in immune responses is a defining feature of the systemic immune response to major injury CNS (i.e. stroke or traumatic brain or spinal cord injury).^10,40-43^ This immunodeficiency resulting from CNS injury contributes significantly to nosocomial infections and resultant mortality.^10,40-43^ Similarly, systemic immune changes that occur with ageing, including expansion of the innate immune compartment and at the expense of adaptive immune cell frequencies, contribute to poor infection clearance and vaccine responses in older adults.^14^ It will be important in future longitudinal experiments to determine if individuals with a low T_H_17 cytokine response are further at risk for infections, poor vaccine responses, and increased instances and severity of delirium, which all decrease quality of life for people with dementia.

In healthy individuals, T_H_17 cells are most abundant in barrier regions like the intestines, where the T_H_17 cells defend against pathogens and support immunity in the mucosa.^44^ The pathogenicity of T_H_17-related inflammation in autoimmune diseases, including multiple sclerosis, is perhaps their most well-known function.^44,45^ A growing body of evidence also finds that systemic inflammatory conditions, such as Type 2 diabetes and healthy ageing, have elevated T_H_17 responses compared with healthy individuals.^25-28,36^ From depression to Parkinson’s disease and beyond, the brain has been described to have signatures of T_H_17-related inflammation.^20,39,46^ This is also true for AD, where prior reports link T_H_17 responses in the brain to AD-related pathology in people and animals.^39,45,47^

Our finding of a blunted T_H_17 response in circulating cells from cognitively impaired individuals may seem paradoxical. Indeed, at first pass, it would be logical to hypothesize that T_H_17 responses should be higher in the blood of people with dementia than in cognitively healthy subjects, particularly as higher T_H_17 responses are seen in healthy ageing.^27,36^ One possible explanation for these seemingly contradictory findings is the neuro-immune reflex arc: a bidirectional communication between the nervous and the immune systems, in which the CNS can regulate inflammatory responses, and the systemic immune system in turn impacts CNS function.^48^ Neuroinflammation may also cause a reflex arc to shut down immune function via direct autonomic innervation. The brain may also release signals into circulation, including damage-associated molecular patterns (DAMPs), such as cell-free DNA. The DAMPs can cause post-injury immunodeficiency independently of direct neuronal innervation.^49^ All the proposed pathways leading to CNS-induced immunodeficiency, including neuroinflammation,^2^ HPA dysfunction,^50^ and release of DAMPs into the circulation,^51^ occur with dementia although the detailed determination of the low T_H_17 signature herein as ‘immunodeficiency’ remains to be demonstrated.

Of the 25 cytokines measured, we found the T_H_17 signature to be the most robust among cytokine families. However, at the individual cytokine level, we also found lower levels of LT-α (also known as TNFβ) and IL-9, in women with cognitive impairment. LT-α is a member of the TNF family of cytokines. Primarily produced by T cells, (T_H_1 and T_H_17), LT-α shares redundant features with TNF-α, acting on TNFRI and RII, but the effects of LT-α are less robust than TNF-α.^32,52^ IL-9, produced by T_H_9 cells, T_H_2 cells Tregs, and T_H_17 cells, was originally defined as a T-cell growth factor, but is now known to have pleiotropic functions in allergy, cancer and autoimmune diseases.^53^ Thus, each of these cytokines can also be produced by T_H_17 cells. While not defining members of the T_H_17 cytokine family, these cytokines likely contribute to the overall loss of T_H_17 function seen in women with cognitive impairment.

The clinical implications of the paucity of CD4/T_H_17 cells in cognitive impairment are unknown. However, respiratory and urinary tract infections are the number 1 and 2 leading causes of hospitalization in older adults with dementia, respectively.^54^ For nursing home residents with advanced dementia, infections are common, as is over/misuse of antimicrobials.^55^ For women, the increased risk of infections may occur years or even a decade before the person loses the ability for self-care. Systemic infections, including respiratory and urinary tract, can also cause delirium.^32^ While the delirium will resolve with the clearance of the infection, it is believed that there may be a lasting effect on the brain that may accelerate cognitive decline.^56,57^ However, much more work is needed to establish a relationship between the T_H_17 signature we observed and dementia.

In this study, we found no association of cognitive impairment with standard biomarkers of AD, including Aβ42/40 ratio, p-tau^181^, NF-L, and GFAP measured in the plasma. These biomarkers, along with p-tau^231^ and p-tau^217^, are excellent at detecting elevated Aβ pathology in the brain even in cognitively healthy individuals.^58^ However, our community-based cohort is not limited to AD and thus may reflect multiple underlying substrates for dementia.^22-24^ In the non-impaired women, a small number of study participants had a low T_H_17 cytokine signature similar to that of the impaired group. Future prospective studies will be critical to test if cognitively healthy women with a low T_H_17 cytokine signature transition into MCI sooner than women sustaining a high T_H_17 cytokine signature. While the *ex vivo* immune stimulation approach will be more difficult to standardize than other fluid biomarkers, if it could identify individuals who will develop dementia, the T_H_17 cytokine signature would be an important tool for early clinical intervention.

It may seem curious that the changes we found in immune function occur primarily in women; however, immune system disparities between the sexes are widely known. For example, almost 80% of autoimmune diseases affect women.^59^ Adult females have greater innate and adaptive immune responses than adult males.^59^ There are numerous reasons why male and female immune systems are distinct. First, the promoters of 50% of the active genes in female T cells include oestrogen response elements.^59^ Additionally, the special function of immunological tolerance during pregnancy plays a part in these variations.^59^ Females often have greater CD4^+^ T-cell counts and CD4/CD8 ratios than age-matched males throughout their lifespan.^59^ While we cannot pinpoint a molecular determinant of the difference in immune response between men and women in our study, it is noteworthy that the female immune system, which is more prone to developing autoimmune diseases and more effective in fighting infections compared with males, is also more likely to experience a decline in women with cognitive impairment.

We note several study limitations. First, the participants from our study are a convenience sample of a single-site ongoing community-based cohort. As reported previously, the cohort is overwhelmingly white race and more educated than the older population in general.^22^ Second, we designed the study to balance CVD risk factors between the groups; therefore, it does not reflect a truly random sampling of the community cohort. Future studies will be needed to replicate our findings in larger samples and across multiple populations, and it will be important to include greater racial and ethnic diversity.

Based on prior studies and our plasma biomarkers, we assume that cognitively impaired individuals have ongoing inflammation in the brain. Future studies using translocator protein 18 kDa (TSPO) PET imaging^60^ are needed for antemortem evaluation of neuroinflammation. The TSPO-PET imaging would directly test the hypothesized neuro-immune reflex arc believed to be responsible for the immunodeficiency associated with cognitive impairment. Ultimately, post-mortem studies are required to determine whether a low T_H_17 response in the blood is correlated with an elevated T_H_17 response in the brain.

## Supplementary material

Supplementary material is available at *Brain Communications* online.

## Supplementary Material

fcad259_Supplementary_Data

## Data Availability

All data generated in this study are available in the main text or the Supplementary material (Supplemental Table 3). Additional data are available following reasonable request to the University of Kentucky Alzheimer Disease Research Center.
