# Supplementary material for: A blunted TH17 cytokine signature in women with mild cognitive impairment: insights from inflammatory profiling of a community-based cohort of older adults
Source: Brain Commun. 2023 Oct 7;5(5):fcad259. doi: 10.1093/braincomms/fcad259 (PMC10612408; doi:10.1093/braincomms/fcad259)
Supplement: fcad259_Supplementary_Data [file fcad259_supplementary_data.pdf]

A blunted T_H17 cytokine signature in women with mild cognitive impairment: insights from inflammatory profiling of a community-based cohort of older adults.

Adam D. Bachstetter[1,2,3,*], Jenny Lutshumba[1,3], Edric Winford[2,] , Erin L Abner[3,5], Barbra J. Martin[3], Jordan P. Harp[3,6], Linda J. Van Eldik[2,3], Frederick A. Schmitt[1,3,6,7], Donna M. Wilcock[2,3,8], Ann M Stowe[2,6], Gregory A. Jicha[3,6], Barbara S. Nikolajczyk[4,9,*]

Supplementary material:

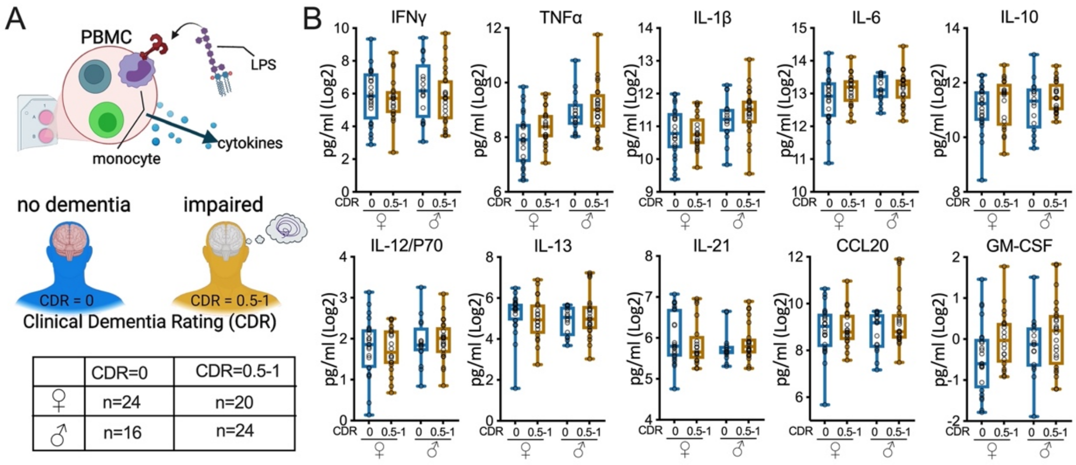

Supplemental Figure 1: Cognitive impairment is not associated with alterations in cytokines produced by LPS-activated PBMCs. (A) PBMCs were plated in a 96 well plate and stimulated with LPS (25ng/ml) for 20 hours. Study participants were divided into healthy controls (CDR=0) or cognitively impaired (CDR=0.5-1). The data was stratified by gender, and there was not significant difference in the number of subjects in the four experimental groups. **(B)** Cytokines produced by the LPS-stimulated PBMCs did not show a different inflammatory profile based on CDR or gender. Statistical tests used: T-test

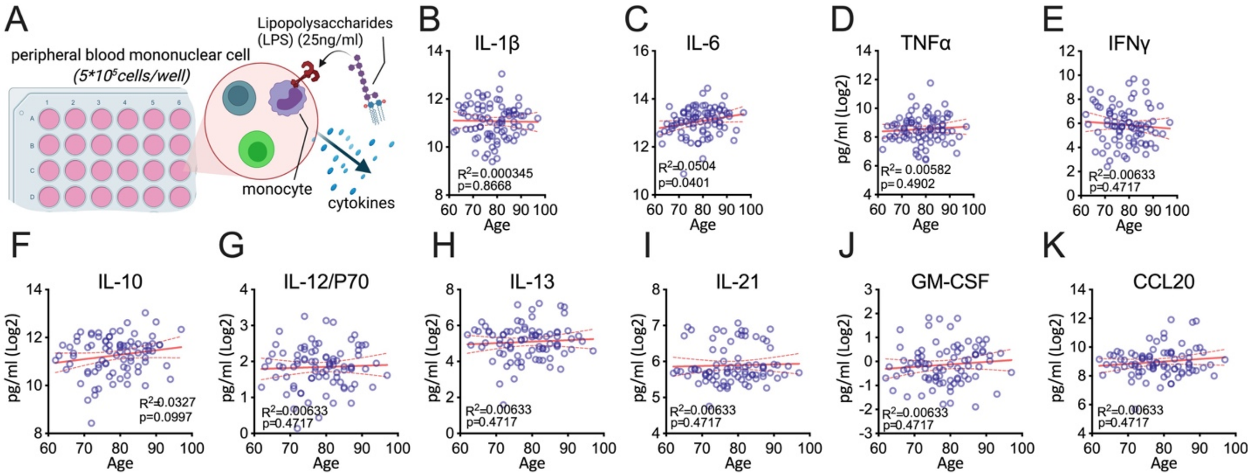

Supplemental Figure 2: Chronological age does not affect the responsiveness of PBMC to LPS stimulation. Aging and LPS. (A) PBMCs were plated in a 96-well plate and stimulated with LPS (25ng/ml) for 20 hours. **(B-K)** No significant association was found between age and the cytokines produced by LPS-stimulated PBMCs. The dashed line shows a 95% confidence interval for the linear regression. p values are uncorrected. Markers are individual participants. Statistical tests used: linear regression.

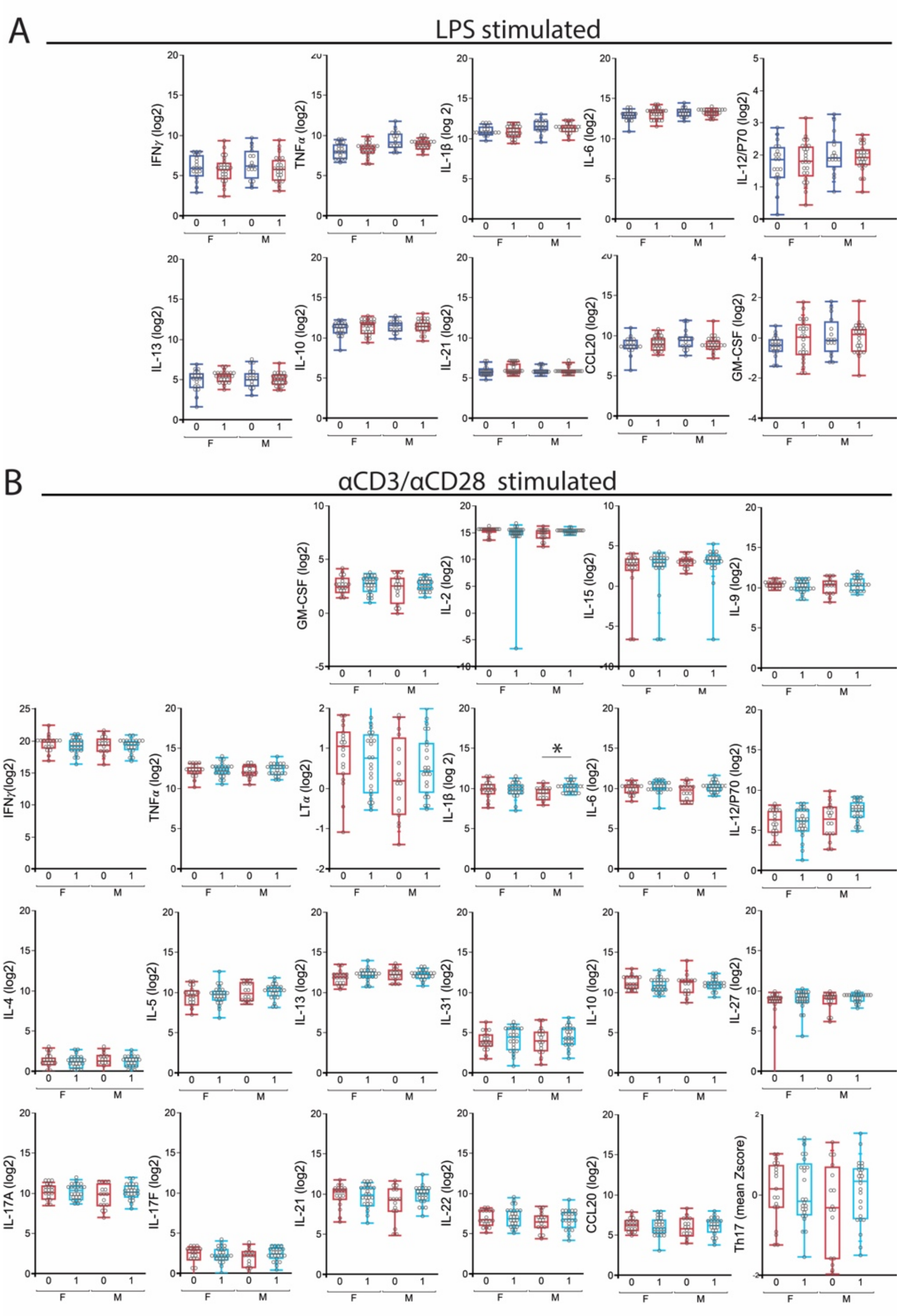

Supplemental Figure 3: The influence of hypertension on cytokine production from stimulated PBMCs. Study participants without hypertension (0) were compared to participants with hypertension (1), and the data was stratified for females (f) and males (m). The cytokines produced by LPS-stimulated **(A)** and αCD3/αCD28 Dynabead stimulated **(B)** PBMCs are shown. Circles are individual participants. *FDR<0.05. See Table S10 for all statistical comparisons. Statistical tests used: T-test

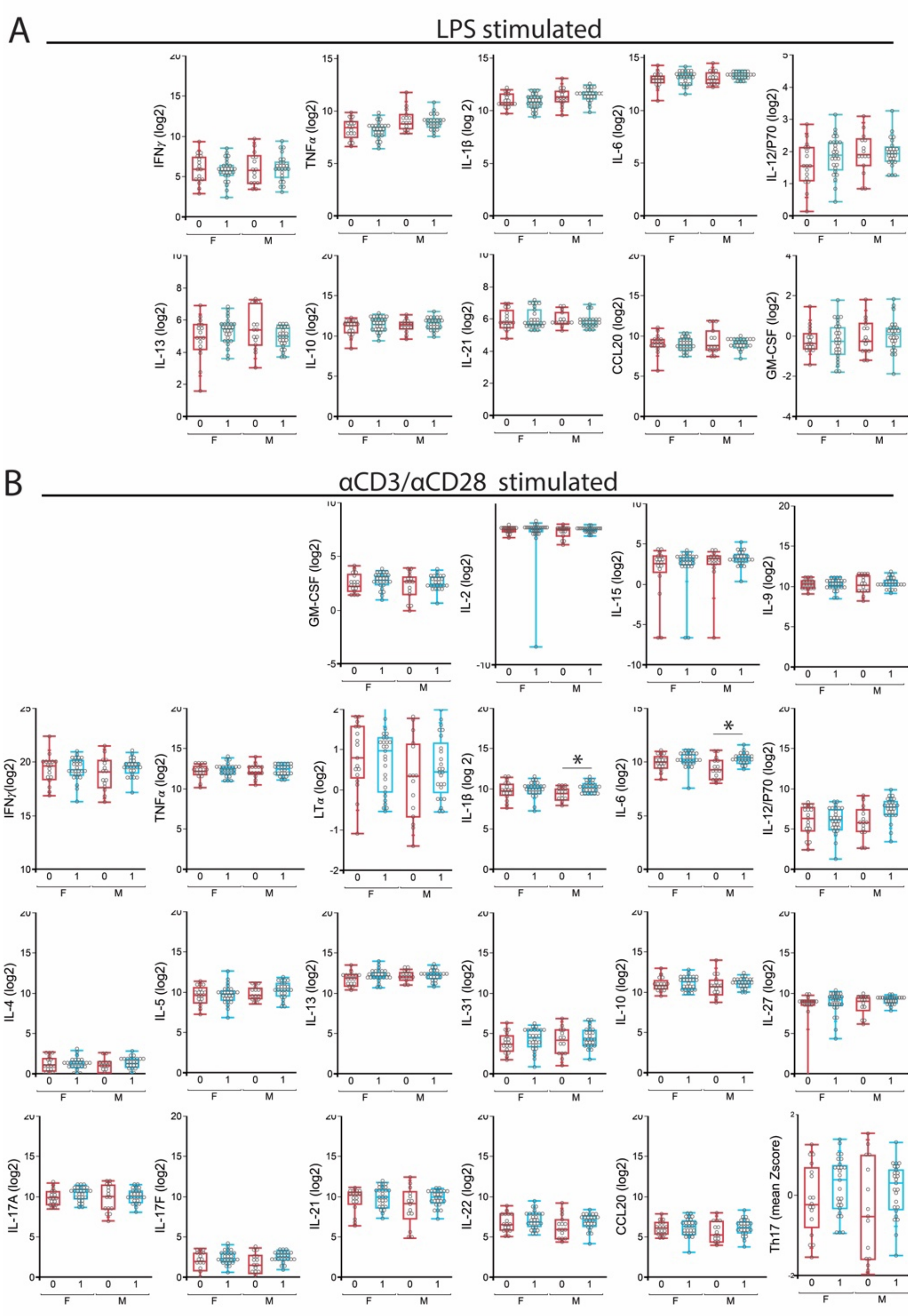

Supplemental Figure 4: The influence hypercholesterolemia on cytokine production from stimulated PBMCs. Study participants without hypercholesterolemia (0) were compared to participants with hypercholesterolemia (1), and the data was stratified for females (f) and males (m). The cytokines produced by LPS-stimulated **(A)** and αCD3/αCD28 Dynabead stimulated **(B)** PBMCs are shown. Circles are individual participants. *FDR<0.05. See Table S10 for all statistical comparisons. Statistical tests used: T-test

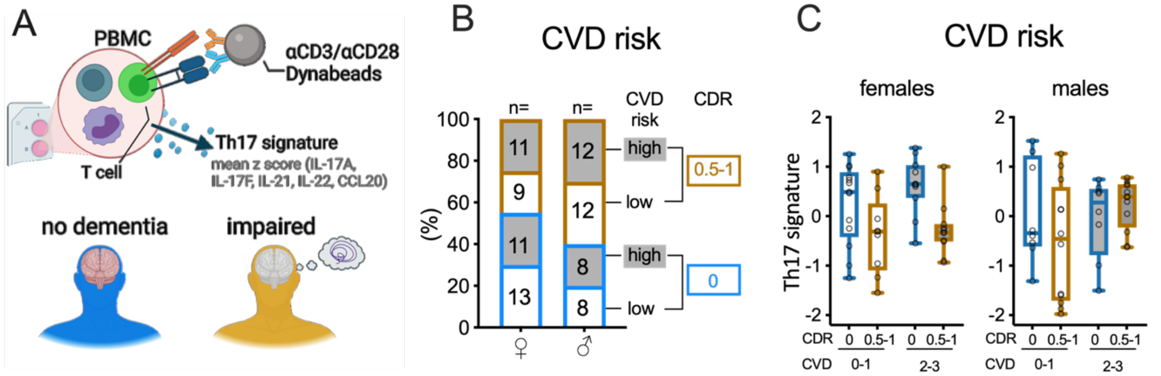

Supplemental Figure 5: Testing the association between cardiovascular disease (CVD) and dementia status. Comparing the dementia status **(A)** between the number of individuals with cardiovascular disease (CVD) risk factors (obesity, hypertension, and hypercholesterolemia) found no difference between experimental groups **(B)** (women, p=0.5454, Chi-square; men, p=1.0, Chi-square). **(C)** Women with cognitive impairment had a decreased T_H17 signature regardless of CVD risk factors, while no difference was found for men (T-test).

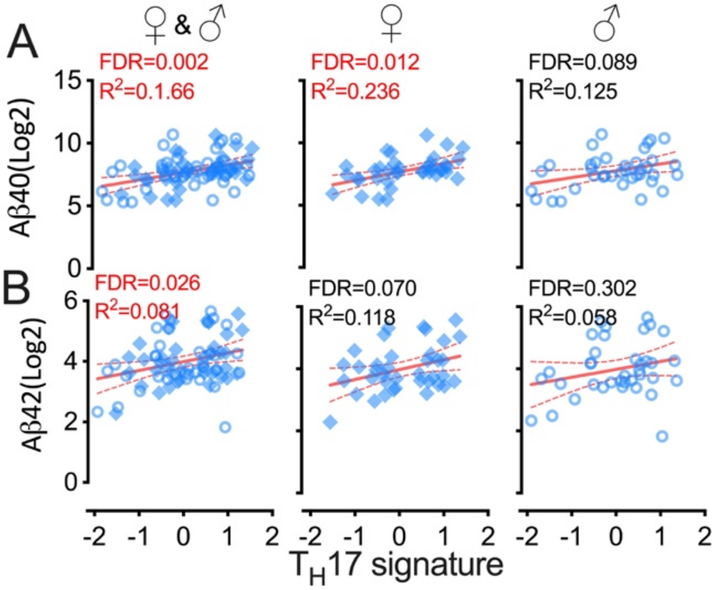

Supplemental Figure 6: The T_H17 cytokine signature correlates with plasma Aβ40 and Aβ42. Plasma Aβ40 **(A)** and Aβ42 **(B)**, measured by Simoa assays, correlated with the T_H17 cytokine signature (mean z score of IL-17A, IL-17F, IL-21, IL-22, CCL20) of the αCD3/αCD28 Dynabead stimulated PBMCs. Open circles are male participants. Closed diamonds are female participants. The dashed line shows 95% confidence interval for the linear regression. p values are for the uncorrected analysis. See also Supplemental Table 13.

		Unstimulated			LPS			αCD3/αCD28		
	**Cytokine**	assay	dilution	detectable	assay	dilution	detectable	assay	dilution	detectable
1	GM-CSF	Bioplex	Neat	no	Bioplex	Neat	yes	Bioplex	Neat	yes
2	IL-1β	Bioplex	Neat	yes	MSD	1:20	yes	Bioplex	Neat	yes
3	IL-2	Bioplex	Neat	no	Bioplex	Neat	no	Bioplex	Neat	yes
4	IL-4	Bioplex	Neat	no	Bioplex	Neat	no	Bioplex	Neat	yes
5	IL-5	Bioplex	Neat	no	Bioplex	Neat	no	Bioplex	Neat	yes
6	IL-6	Bioplex	Neat	yes	MSD	1:20	yes	Bioplex	Neat	yes
7	IL-9	Bioplex	Neat	no	Bioplex	Neat	no	Bioplex	Neat	yes
8	IL-10	Bioplex	Neat	yes	Bioplex	Neat	yes	Bioplex	Neat	yes
9	IL-12/P70	Bioplex	Neat	no	Bioplex	Neat	yes	Bioplex	Neat	yes
10	IL-13	Bioplex	Neat	no	Bioplex	Neat	yes	Bioplex	Neat	yes
11	IL-15	Bioplex	Neat	no	Bioplex	Neat	yes	Bioplex	Neat	yes
12	IL-17A	Bioplex	Neat	no	Bioplex	Neat	no	MSD	1:10	yes
13	IL-17F	Bioplex	Neat	no	Bioplex	Neat	no	Bioplex	Neat	yes
14	IL-17E	Bioplex	Neat	no	Bioplex	Neat	no	Bioplex	Neat	no
15	IL-21	Bioplex	Neat	no	Bioplex	Neat	yes	MSD	1:10	yes
16	IL-22	Bioplex	Neat	no	Bioplex	Neat	no	MSD	1:10	yes
17	IL-23	Bioplex	Neat	no	Bioplex	Neat	no	MSD	1:10	yes
18	IL-27	Bioplex	Neat	no	Bioplex	Neat	no	MSD	1:10	yes
19	IL-28	Bioplex	Neat	no	Bioplex	Neat	no	Bioplex	Neat	no
20	IL-31	Bioplex	Neat	no	Bioplex	Neat	no	MSD	1:10	yes
21	IL-33	Bioplex	Neat	no	Bioplex	Neat	no	Bioplex	Neat	no
22	CCL20	Bioplex	Neat	no	Bioplex	Neat	yes	MSD	1:10	yes
23	TNFα	Bioplex	Neat	yes	MSD	1:20	yes	MSD	1:5000	yes
24	LTα	Bioplex	Neat	no	Bioplex	Neat	no	Bioplex	Neat	yes
25	IFNγ	Bioplex	Neat	yes	MSD	1:20	yes	MSD	1:8000	yes

Supplemental Table 1: Summary of the cytokine assays. All the cytokines were first measured with Bioplex using the conditioned media that was undiluted. For those cytokines that were out of the dynamic range of the Bioplex assay, they were diluted and rerun on the Mesoscale Discovery Assay (MSD).

	Cytokine	Company	Catalog no.	LLOQ (pg/ml)	ULOQ (pg/ml)
1	GM-CSF	Bioplex		162.9	131600
2	IL-1β	Bioplex		4.506	20393.6
3	IL-2	Bioplex		54	45202.1
4	IL-4	Bioplex		826	100083.8
5	IL-5	Bioplex		2.338375	25357.08
6	IL-6	Bioplex		66.8	9800.5
7	IL-9	Bioplex		33.3	34143.2
8	IL-10	Bioplex		30.5	5243.3
9	IL-12/P70	Bioplex		7.2	20276.7
10	IL-13	Bioplex		89.4	29945.7
11	IL-15	Bioplex		41.1	20414.1
12	IL-17A	Bioplex		48.3	53684.3
13	IL-17F	Bioplex		19.3	106128.8
14	IL-17E	Bioplex		41.3	43864
15	IL-21	Bioplex		41.0	20621.5
16	IL-22	Bioplex		0.11	150.2
17	IL-23	Bioplex		1198.4	1524056.3
18	IL-27	Bioplex		390.6	260395
19	IL-28	Bioplex		1792	521332.5
20	IL-31	Bioplex		14.9	192796
21	IL-33	Bioplex		4.5	20349.8
22	CCL20	Bioplex		82.4	10746.4
23	TNFα	Bioplex	HT17MG-	14.1	8829.4
24	LTα	Bioplex	14K-PX25	0.09	44.9
25	IFNγ	Bioplex		100.6	40356.6
26	IL-1β	MSD		0.64	375
27	IL-6	MSD		0.63	488
28	TNFα	MSD	K15052D	0.69	248
29	IFNγ	MSD		1.76	936
30	IL-17A	MSD	K15085D	5.85	1950
31	IL-21	MSD		6.13	650
32	IL-22	MSD		2.78	325
33	IL-23	MSD		4.60	3250
34	IL-27	MSD		38.7	13000
35	IL-31	MSD		4.22	650
36	CCL20	MSD		0.880	325

Supplemental Table 2: Summary of the cytokine assays dynamic range. Lower limit of quantification (LLOQ). Limit of detection (LOD), Upper limit of quantification (ULOQ).

				CDR=0				CDR=0.5-1			
				Women		Men		Women		Men	
	cytokine	units	assay	mean	SD	mean	SD	mean	SD	mean	SD
LPS stimulated	IFNγ	pg/ml	MSD	114.5	140.3	156.9	204.1	68.7	75.7	103.1	160.6
	TNFα	pg/ml	MSD	343.3	226.1	771.4	760.8	328.1	172.3	487.9	179.9
	IL-1β	pg/ml	MSD	2049.4	886.4	3129.9	1792.1	1929.5	907.2	2722.2	928.4
	IL-6	pg/ml	MSD	8152.3	3683.1	9632.4	4140.2	9290.9	3571.9	9827.2	2124.5
	IL-12/P70	pg/ml	Bioplex	3.5	1.5	4.3	2.0	3.9	1.6	3.9	1.1
	IL-13	pg/ml	Bioplex	38.8	26.9	53.8	46.2	46.0	22.2	30.6	12.5
	IL10	pg/ml	Bioplex	2524.0	1171.5	2643.3	1265.5	2862.9	1545.2	3236.4	1697.6
	IL-21	pg/ml	Bioplex	63.0	27.7	62.8	21.8	70.6	31.6	57.6	19.1
	CCL20	pg/ml	Bioplex	587.5	441.0	1023.0	1159.9	574.6	329.7	471.5	170.4
	GM-CSF	ng/ml	Bioplex	0.9	0.5	1.3	0.8	1.1	0.7	1.1	0.7
αCD3/αCD28 stimulated	GM-CSF	ng/ml	Bioplex	6.4	4.0	6.6	4.3	7.0	2.9	6.0	2.1
	IL-2	ng/ml	Bioplex	33.6	10.8	31.6	15.5	35.9	16.8	36.5	10.3
	IL-15	pg/ml	Bioplex	7.4	5.4	8.2	3.9	7.6	3.6	11.5	7.8
	IL-9	pg/ml	Bioplex	1334.5	483.0	1544.9	802.7	1233.0	550.8	1324.6	695.1
	IFNγ	ng/ml	MSD	951.3	1079.8	791.3	721.3	801.9	514.2	808.0	437.2
	TNFα	pg/ml	MSD	4890.3	2174.6	5514.3	3114.7	5946.1	3411.5	5164.2	2294.6
	LTα	pg/ml	Bioplex	2.0	0.9	1.6	1.0	1.8	1.0	1.6	0.9
	IL-1β	pg/ml	Bioplex	1089.8	676.0	767.6	357.5	1046.4	584.4	1289.5	536.8
	IL-6	pg/ml	Bioplex	1056.2	413.8	874.2	503.5	1278.4	487.5	1320.2	559.0
	IL-12/P70	pg/ml	Bioplex	103.4	89.1	163.4	222.5	100.9	90.8	226.4	153.8
	IL-4	ng/ml	Bioplex	2.9	1.9	2.9	1.8	2.5	1.3	2.3	0.9
	IL-5	pg/ml	Bioplex	1000.6	747.2	1422.4	901.4	1061.6	1208.3	1246.4	826.2
	IL-13	pg/ml	Bioplex	4291.0	2648.5	5254.7	2346.7	5054.4	2907.8	4380.5	1672.3
	IL-31	pg/ml	MSD	20.3	17.7	34.3	33.9	26.7	20.0	24.6	19.1
	IL-10	pg/ml	Bioplex	2552.0	1634.6	2723.6	3321.7	2345.7	1493.0	2114.7	897.8
	IL-27	pg/ml	MSD	494.0	232.4	507.9	244.1	543.1	289.3	578.4	170.7
	IL-17A	pg/ml	MSD	1304.2	872.6	1320.6	1193.2	1365.8	664.6	1268.5	650.7
	IL-17F	pg/ml	MSD	5.2	3.4	4.7	3.5	6.1	3.5	6.2	2.4
	IL-21	pg/ml	MSD	1130.6	787.3	1136.7	1352.5	1157.6	791.0	1006.7	529.4
	IL-22	pg/ml	MSD	145.6	111.2	126.3	138.7	181.1	159.1	136.5	64.9
	IL-23	pg/ml	MSD	175.0	148.7	131.2	113.7	180.2	129.4	245.0	164.0
	CCL20	pg/ml	MSD	83.4	49.2	82.4	83.9	90.0	67.2	81.7	40.3

Supplemental Table 3. Summary of cytokine concentrations in the media of LPS or from αCD3/αCD28 stimulated PBMCs.

		women					
		unadjusted		model 1		model 2	
		FDR=	d[1]	FDR=	d[1]	FDR=	d[1]
LPS stimulated	IFNγ	0.903	0.248	0.968	0.221	0.206	0.986
	TNFα	0.367	0.581	0.388	0.608	0.6	0.82
	IL-1β	0.935	0.021	0.987	0.024	0.02	0.986
	IL-6	0.367	0.409	0.656	0.327	0.322	0.942
	IL-10	0.903	0.064	0.968	0.074	0.078	0.986
	IL-12/P70	0.853	0.287	0.898	0.34	0.349	0.947
	IL-13	0.853	0.227	0.968	0.126	0.124	0.986
	IL-21	0.853	0.181	0.898	0.178	0.182	0.97
	CCL-20	0.903	0.088	0.987	0.006	0.007	0.986
	GM-CSF	0.367	0.605	0.388	0.571	0.562	0.82
αCD3/αCD28 stimulated	GM-CSF	0.069	0.72	0.116	0.648	0.193	0.674
	IL-2	0.642	0.903	0.657	1.033	0.777	1.082
	IL-15	0.823	0.462	0.933	0.222	0.978	0.228
	IL-9	**0.008**	0.835	**0.021**	0.758	**0.048**	0.775
	IFNγ	0.247	0.74	0.43	0.591	0.647	0.611
	TNFα	0.91	0.073	0.933	0.028	0.978	0.016
	LTα	**0.008**	1.055	**0.021**	0.932	**0.048**	0.957
	IL-1β	0.621	0.347	0.566	0.388	0.747	0.395
	IL-6	0.959	0.032	0.933	0.03	0.978	0.023
	IL-12/P70	0.208	1.145	0.303	1.039	0.481	1.07
	IL-4	0.642	0.261	0.756	0.181	0.858	0.185
	IL-5	0.642	0.312	0.657	0.331	0.777	0.338
	IL-13	0.969	0.013	0.933	0.073	0.978	0.083
	IL-31	0.642	0.368	0.657	0.388	0.777	0.407
	IL-10	0.356	0.433	0.474	0.385	0.676	0.393
	IL-27	0.147	1.687	0.123	1.82	0.246	1.877
	IL-17A	**0.028**	1.033	**0.03**	1.072	0.058	1.105
	IL-17F	0.11	1.008	0.095	1.098	0.164	1.14
	IL-21	**0.008**	1.833	**0.021**	1.752	**0.048**	1.805
	IL-22	**0.042**	1.145	**0.039**	1.199	0.074	1.238
	IL-23	0.186	0.982	0.272	0.902	0.446	0.934
	CCL20	**0.028**	1.109	**0.03**	1.142	**0.066**	1.169

Supplemental Table 4. Association of CDR in women and cytokines produced by stimulated PBMCs. Model 1 is a linear regression adjusted for age. Model 2 is a linear regression adjusted for age and 0-1 vs. 2-3 cardiovascular disease (CVD) risk factors (obesity, hypertension, and hypercholesterolemia). Benjamini-Hochberg false discover rate (FDR) adjusted p values. [1]Cohen's d. Bolded Red font highlights statistically significant results.

		men					
		unadjusted		model 1		model 2	
		FDR=	d[1]	FDR=	d[1]	FDR=	d[1]
LPS stimulated	IFNγ	0.608	0.559	0.642	0.522	0.732	0.521
	TNFα	0.717	0.194	0.734	0.187	0.732	0.18
	IL-1β	0.608	0.285	0.642	0.299	0.638	0.295
	IL-6	0.771	0.055	0.799	0.048	0.837	0.049
	IL-10	0.771	0.065	0.799	0.064	0.837	0.062
	IL-12/P70	0.608	0.307	0.642	0.296	0.732	0.285
	IL-13	0.608	0.363	0.642	0.351	0.638	0.352
	IL-21	0.608	0.171	0.642	0.16	0.678	0.157
	CCL-20	0.608	0.544	0.642	0.528	0.638	0.513
	GM-CSF	0.608	0.362	0.642	0.355	0.638	0.35
αCD3/αCD28 stimulated	GM-CSF	0.777	0.161	0.804	0.152	0.767	0.157
	IL-2	0.777	0.182	0.804	0.177	0.706	0.185
	IL-15	0.7	1.1	0.719	1.082	0.287	1.075
	IL-9	0.7	0.415	0.719	0.396	0.422	0.397
	IFNγ	0.966	0.035	0.909	0.065	0.905	0.054
	TNFα	0.777	0.115	0.804	0.115	0.767	0.115
	LTα	0.777	0.146	0.804	0.126	0.767	0.131
	IL-1β	0.966	0.012	0.942	0.02	0.98	0.005
	IL-6	0.835	0.09	0.853	0.084	0.803	0.097
	IL-12/P70	0.777	0.393	0.804	0.385	0.71	0.366
	IL-4	0.7	0.586	0.719	0.574	0.204	0.573
	IL-5	0.777	0.377	0.789	0.382	0.519	0.379
	IL-13	0.7	0.32	0.719	0.321	0.425	0.318
	IL-31	0.7	0.75	0.719	0.75	0.412	0.751
	IL-10	0.777	0.316	0.804	0.284	0.557	0.29
	IL-27	0.7	0.397	0.732	0.383	0.425	0.393
	IL-17A	0.822	0.156	0.853	0.125	0.803	0.137
	IL-17F	0.777	0.34	0.804	0.31	0.586	0.328
	IL-21	0.777	0.5	0.804	0.47	0.612	0.485
	IL-22	0.777	0.235	0.804	0.206	0.706	0.221
	IL-23	0.727	0.527	0.758	0.521	0.434	0.506
	CCL20	0.777	0.284	0.804	0.255	0.706	0.265

Supplemental Table 5. Association of CDR in men and cytokines produced stimulated PBMCs. Model 1 is a multiple linear regression adjusted for age. Model 2 is a multiple linear regression adjusted for age and 0-1 vs. 2-3 cardiovascular disease (CVD) risk factors (obesity, hypertension, and hypercholesterolemia). Benjamini-Hochberg false discover rate (FDR) adjusted p values. [1]Cohen's d. Bolded Red font highlights statistically significant results.

	women						men					
	unadjusted		model 1		model 2		unadjusted		model 1		model 2	
	p=	d[1]	p=	d[1]	p=	d[1]	p=	d[1]	p=	d[1]	p=	d[1]
T_H17 signature v. CDR	**<0.001**	0.775	**<0.001**	0.832	**<0.001**	0.874	0.584	0.178	0.6484	0.149	0.652	0.147

Supplemental Table 6. Association of clinical dementia rating (CDR) score and the mean T_H17 cytokine z-score from αCD3/αCD28 PBMCs (T_H17 signature). Model 1 is adjusted for age. Model 2 is adjusted for age and 0-1 vs. 2-3 cardiovascular disease (CVD) risk factors (obesity, hypertension, and hypercholesterolemia). [1]Cohen's d. Bolded Red font highlights statistically significant results.

		Aging					
		both		women		men	
		FDR=	R^2	FDR=	R^2	FDR=	R^2
LPS stimulated	IFNγ	0.755	0.006	0.973	0.002	0.940	0.019
	TNFα	0.755	0.006	0.973	0.000	0.940	0.002
	IL-1β	0.867	0.000	0.973	0.000	0.940	0.030
	IL-6	0.221	0.050	0.708	0.074	0.940	0.010
	IL-12/P70	0.830	0.002	0.973	0.001	0.940	0.000
	IL-13	0.755	0.004	0.973	0.007	0.940	0.004
	IL10	0.365	0.033	0.708	0.054	0.940	0.007
	IL-21	0.830	0.001	0.973	0.001	0.940	0.028
	CCL20	0.620	0.018	0.746	0.029	0.940	0.006
	GM-CSF	0.755	0.010	0.914	0.016	0.940	0.001
αCD3/αCD28 stimulated	GM-CSF	0.264	0.031	0.570	0.058	0.791	0.010
	IL-2	0.882	0.001	0.989	0.002	0.834	0.004
	IL-15	0.264	0.028	0.698	0.024	0.683	0.018
	IL-9	**0.044**	0.083	0.401	0.100	0.191	0.084
	IFNγ	**0.025**	0.100	0.565	0.073	0.118	0.132
	TNFα	0.631	0.009	0.570	0.053	0.834	0.004
	LTα	**0.018**	0.128	0.229	0.146	0.191	0.088
	IL-1β	0.882	0.001	0.989	0.002	0.683	0.019
	IL-6	0.712	0.006	0.989	0.000	0.683	0.016
	IL-12/P70	0.903	0.000	0.698	0.027	0.834	0.003
	IL-4	0.160	0.046	0.678	0.036	0.303	0.057
	IL-5	0.717	0.005	0.989	0.000	0.796	0.008
	IL-13	0.631	0.009	0.766	0.016	0.902	0.000
	IL-31	0.882	0.001	0.989	0.000	0.895	0.001
	IL-10	**0.023**	0.110	0.698	0.021	**0.025**	0.245
	IL-27	0.924	0.000	0.989	0.002	0.323	0.052
	IL-17A	0.184	0.040	0.989	0.000	0.163	0.110
	IL-17F	0.343	0.021	0.989	0.004	0.118	0.133
	IL-21	0.100	0.062	0.570	0.047	0.266	0.068
	IL-22	0.264	0.027	0.989	0.000	0.191	0.086
	IL-23	0.815	0.003	0.698	0.026	0.834	0.003
	CCL20	0.160	0.046	0.989	0.001	0.118	0.141

Supplemental Table 7. Association of age and cytokines produced by stimulated PBMCs. Results of linear regression an Benjamini-Hochberg false discover rate (FDR) adjusted p values. [1]Cohen's d. Bolded Red font highlights statistical differences.

		APOE					
		both		women		men	
		FDR=	d[1]	FDR=	d[1]	FDR=	d[1]
LPS stimulated	IFNγ	0.551	0.381	0.197	1.184	0.691	0.711
	TNFα	0.515	0.286	0.981	0.008	**0.050**	0.993
	IL-1β	0.980	0.005	0.126	0.606	**0.022**	1.007
	IL-6	0.551	0.143	0.281	0.485	0.189	0.373
	IL-12/P70	0.515	0.183	0.968	0.024	0.123	0.479
	IL-13	0.425	0.459	0.968	0.032	0.031	1.118
	IL10	0.533	0.217	0.780	0.173	0.733	0.219
	IL-21	0.964	0.012	0.355	0.311	0.168	0.359
	CCL20	0.571	0.222	0.480	0.427	**0.043**	1.135
	GM-CSF	0.894	0.058	0.689	0.224	0.733	0.277
αCD3/αCD28 stimulated	GM-CSF	0.485	0.293	0.968	0.037	0.189	0.645
	IL-2	0.483	1.099	0.469	1.616	0.680	0.308
	IL-15	0.485	0.846	0.355	1.480	0.733	0.270
	IL-9	0.515	0.193	0.355	0.355	0.985	0.028
	IFNγ	0.515	0.345	0.281	0.758	0.931	0.172
	TNFα	0.894	0.041	0.820	0.119	0.985	0.030
	LTα	0.392	0.381	0.126	0.692	0.975	0.094
	IL-1β	0.358	0.494	0.126	0.852	0.985	0.007
	IL-6	0.808	0.093	0.532	0.253	0.975	0.091
	IL-12/P70	0.358	0.921	0.483	0.714	0.543	0.801
	IL-4	0.515	0.239	0.731	0.189	0.056	0.812
	IL-5	0.358	0.559	0.968	0.045	0.031	1.218
	IL-13	0.515	0.211	0.526	0.288	0.026	0.828
	IL-31	0.515	0.399	0.355	0.729	0.022	1.775
	IL-10	0.865	0.067	0.780	0.140	0.931	0.127
	IL-27	0.323	1.115	0.355	1.436	0.267	0.642
	IL-17A	0.865	0.092	0.658	0.262	0.985	0.023
	IL-17F	0.660	0.223	0.500	0.490	0.985	0.070
	IL-21	0.443	0.629	0.355	0.818	0.733	0.497
	IL-22	0.515	0.353	0.126	1.009	0.789	0.313
	IL-23	0.323	0.771	0.355	0.830	0.497	0.620
	CCL20	0.323	0.658	**0.049**	1.162	0.975	0.111

Supplemental Table 8. Association of *apoE4* and cytokines produced by stimulated PBMCs. Results of T-test and Benjamini-Hochberg false discover rate (FDR) adjusted p values. [1]Cohen's d. Bolded Red font highlights statistical differences.

		CVD risk			
		women		men	
		FDR=	d[1]	FDR=	d[1]
LPS stimulated	IFNγ	0.742	0.616	0.667	0.148
	TNFα	0.857	0.059	0.5	0.356
	IL-1β	0.742	0.16	0.727	0.693
	IL-6	0.742	0.261	0.667	0.109
	IL-12/P70	0.742	0.234	0.708	0.605
	IL-13	0.742	0.578	0.5	1.94
	IL10	0.742	0.203	0.5	0.136
	IL-21	0.742	0.193	0.667	0.491
	CCL20	0.834	0.109	0.5	0.095
	GM-CSF	0.742	0.218	0.708	0.127
αCD3/αCD28 stimulated	GM-CSF	0.975	0.266	0.56	0.399
	IL-2	0.975	1.092	0.228	0.591
	IL-15	0.975	0.39	0.407	1.063
	IL-9	0.975	0.235	0.819	0.269
	IFNγ	0.975	0.091	0.407	0.081
	TNFα	0.975	0.33	0.851	1.383
	LTα	0.975	0.163	0.72	0.273
	IL-1β	0.975	0.055	**0.006**	0.852
	IL-6	0.975	0.329	**0.032**	0.109
	IL-12/P70	0.997	0.003	0.129	0.922
	IL-4	0.975	0.166	0.534	0.474
	IL-5	0.985	0.034	0.72	0.258
	IL-13	0.975	0.417	0.407	0.191
	IL-31	0.975	0.377	0.876	0.786
	IL-10	0.975	0.145	0.851	0.164
	IL-27	0.975	0.612	0.407	0.429
	IL-17A	0.975	0.245	0.407	0.078
	IL-17F	0.975	0.678	0.141	0.058
	IL-21	0.975	0.164	0.407	0.492
	IL-22	0.975	0.369	0.228	0.889
	IL-23	0.975	0.18	**0.032**	0.512
	CCL20	0.975	0.087	0.51	0.685

Supplemental Table 9. Association of (CVD) risk factors (obesity, hypertension, and hypercholesterolemia) and cytokines produced by stimulated PBMCs. Results of T-test and Benjamini-Hochberg false discover rate (FDR) adjusted p values. [1]Cohen's d. Bolded Red font highlights statistical differences.

		hypertension				hypercholesterolemia			
		women		men		women		men	
		FDR=	d[1]	FDR=	d[1]	FDR=	d[1]	FDR=	d[1]
LPS stimulated	IFNγ	0.807	0.25	0.633	0.764	0.834	0.312	0.928	0.065
	TNFα	0.643	0.29	0.150	0.727	0.827	0.226	0.761	0.181
	IL-1β	0.807	0.062	0.633	0.314	0.827	0.196	0.761	0.252
	IL-6	0.485	0.35	0.633	0.144	0.827	0.221	0.49	0.371
	IL-12/P70	0.807	0.075	0.633	0.17	0.81	0.336	0.84	0.066
	IL-13	0.485	0.557	0.633	0.32	0.81	0.543	0.761	0.505
	IL10	0.794	0.199	0.945	0.025	0.827	0.319	0.761	0.294
	IL-21	0.485	0.329	0.945	0.012	0.932	0.031	0.761	0.101
	CCL20	0.643	0.329	0.150	0.913	0.928	0.118	0.761	0.293
	GM-CSF	0.485	0.401	0.775	0.163	0.932	0.027	0.761	0.179
αCD3/αCD28 stimulated	GM-CSF	0.942	0.057	0.596	0.395	0.721	0.233	0.304	0.492
	IL-2	0.894	1.06	0.126	0.733	0.757	0.821	0.191	0.544
	IL-15	0.894	0.447	0.91	0.223	0.716	0.927	0.29	1.036
	IL-9	0.894	0.371	0.892	0.161	0.773	0.134	0.397	0.318
	IFNγ	0.894	0.462	0.91	0.146	0.972	0.028	0.191	0.84
	TNFα	0.894	0.181	0.712	0.225	0.694	0.263	0.539	0.182
	LTα	0.894	0.224	0.701	0.304	0.838	0.125	0.304	0.455
	IL-1β	0.942	0.048	**0.01**	1.003	0.876	0.116	**0.002**	1.14
	IL-6	0.894	0.235	0.184	0.676	0.563	0.354	**0.021**	1
	IL-12/P70	0.894	0.227	0.147	1.593	0.972	0.022	0.056	1.867
	IL-4	0.894	0.182	0.91	0.049	0.972	0.019	0.383	0.355
	IL-5	0.942	0.065	0.91	0.045	0.972	0.024	0.338	0.495
	IL-13	0.894	0.392	0.91	0.06	0.502	0.5	0.606	0.137
	IL-31	0.894	0.256	0.596	0.6	0.694	0.497	0.397	0.57
	IL-10	0.894	0.447	0.91	0.053	0.773	0.163	0.407	0.353
	IL-27	0.894	0.973	0.284	0.588	0.721	0.618	0.13	0.713
	IL-17A	0.953	0.021	0.596	0.544	0.548	0.535	0.383	0.504
	IL-17F	0.894	0.389	0.354	0.744	0.451	0.813	0.068	1.181
	IL-21	0.894	0.192	0.248	1.226	0.721	0.385	0.191	1.163
	IL-22	0.894	0.222	0.871	0.274	0.694	0.409	0.076	1.046
	CCL20	0.894	0.283	0.892	0.215	0.876	0.136	0.271	0.699

Supplemental Table 10. Association of hypertension and hypercholesterolemia and cytokines produced by stimulated PBMCs. Results of T-test and Benjamini-Hochberg false discover rate (FDR) adjusted p values. [1]Cohen's d. Bolded Red font highlights statistical differences.

		BMI					
		both		women		men	
		FDR=	R^2	FDR=	R^2	FDR=	R^2
LPS stimulated	IFNγ	0.346	0.055	0.238	0.094	0.878	0.032
	TNFα	0.422	0.027	0.551	0.023	0.300	0.122
	IL-1β	0.897	0.000	0.623	0.008	0.930	0.000
	IL-6	0.875	0.002	0.551	0.021	0.878	0.006
	IL-12/P70	0.682	0.007	0.574	0.016	0.878	0.005
	IL-13	0.682	0.009	0.574	0.013	0.878	0.005
	IL10	0.875	0.002	0.551	0.026	0.878	0.015
	IL-21	0.682	0.010	0.551	0.035	0.878	0.007
	CCL20	0.422	0.025	0.551	0.043	0.878	0.015
	GM-CSF	0.875	0.001	0.832	0.001	0.878	0.003
αCD3/αCD28 stimulated	GM-CSF	0.954	0.000	0.992	0.006	0.828	0.006
	IL-2	0.954	0.000	0.997	0.001	0.926	0.001
	IL-15	0.917	0.011	0.958	0.011	0.910	0.002
	IL-9	0.917	0.012	0.958	0.008	0.548	0.023
	IFNγ	0.954	0.001	0.997	0.000	0.828	0.007
	TNFα	0.954	0.000	0.997	0.000	0.910	0.002
	LTα	0.954	0.001	0.958	0.012	0.970	0.000
	IL-1β	0.954	0.001	0.598	0.060	0.378	0.092
	IL-6	0.917	0.007	0.997	0.002	0.440	0.061
	IL-12/P70	0.954	0.002	0.958	0.009	0.548	0.030
	IL-4	0.917	0.008	0.997	0.000	0.460	0.045
	IL-5	0.954	0.001	0.997	0.000	0.548	0.025
	IL-13	0.917	0.017	0.997	0.000	0.378	0.129
	IL-31	0.954	0.003	0.958	0.014	0.378	0.087
	IL-10	0.954	0.000	0.958	0.025	0.585	0.019
	IL-27	0.104	0.072	0.598	0.070	0.378	0.092
	IL-17A	0.104	0.079	0.070	0.164	0.494	0.039
	IL-17F	0.060	0.104	0.070	0.165	0.440	0.051
	IL-21	0.917	0.007	0.958	0.025	0.926	0.001
	IL-22	0.443	0.037	0.910	0.038	0.440	0.053
	IL-23	0.917	0.006	0.997	0.002	0.440	0.059
	CCL20	0.917	0.014	0.958	0.010	0.548	0.026

Supplemental Table 11. Association of body mass index (BMI) and cytokines produced by stimulated PBMCs. Results of linear regression an Benjamini-Hochberg false discover rate (FDR) adjusted p values. Bolded Red font highlights statistically significant results.

		both			women			men		
		F=	p=	d[1]	F=	p=	d[1]	F=	p=	d[1]
CDR+MMSE	overall	9.66	**0.0002**		4.99	**0.0121**		7.41	**0.002**	
	Control v MCI		0.838	0.14		0.0784	0.84		0.629	0.32
	Control v dementia		**0.0001**	1.38		**0.0254**	1.2		**0.009**	1.52
	MCI v. dementia		**0.0013**	1.24		0.7523	0.36		**0.002**	1.84
consensus	overall	2.64	0.078		3.93	**0.0284**		0.29	0.749	
	Control v MCI		0.6418	0.22		0.1963	0.64		0.936	0.12
	Control v dementia		0.0628	0.72		**0.0378**	1.13		0.883	0.22
	MCI v. dementia		0.2801	0.5		0.5758	0.5		0.73	0.34

Supplemental Table 12. Association of mean T_H17 cytokine z-score from αCD3/αCD28 PBMCs (T_H17 signature) and cognitive impairment. The clinical dementia rating (CDR) and Mini-Mental State Examination (MMSE) scores were combined (CDR+MMSE) to define control (CDR=0, MMSE ≥ 25), mild cognitive impairment (MCI) (CDR=0.5, MMSE ≥ 25), and dementia (CDR=0.5 and MMSE score <25, or CDR=1). Neuropsychological consensus agreement among the UK-ADRC neuropsychologists was also used to define control, MCI or dementia status. Pairwise comparison used the Tukey test. Results are adjusted for age and education. [1]Cohen's d. Bolded Red font highlights statistical differences.

Plasma biomarkers		PBMC T_H17 signature					
		both		women		men	
		FDR=	R^2	FDR=	R^2	FDR=	R^2
Aβ42/Aβ40	unadjusted	0.077	0.051	0.0869	0.096	0.5759	0.022
	age adj	0.163	0.083	0.0986	0.102	0.935	0.15
Aβ40 (log2)	unadjusted	**0.002**	0.166	**0.0118**	0.236	0.0891	0.125
	age adj	**0.009**	0.216	**0.0147**	0.261	0.498	0.226
Aβ42 (log2)	unadjusted	**0.026**	0.081	0.0701	0.118	0.3015	0.058
	age adj	0.052	0.095	0.0829	0.135	0.498	0.073
p-tau^{181} (log2)	unadjusted	0.84	<0.001	0.9448	<0.001	0.8283	0.002
	age adj	0.868	0.001	0.9531	0.004	0.935	0.018
NF-L (log2)	unadjusted	**0.015**	0.1	0.1149	0.07	0.0891	0.141
	age adj	0.052	0.305	0.1208	0.18	0.498	0.512
GFAP (log2)	unadjusted	0.138	0.037	**0.0307**	0.179	0.8283	0.002
	age adj	0.413	0.249	**0.0147**	0.416	0.498	0.364

Supplemental Table 13. Association of T_H17-associated cytokines from αCD3/αCD28 stimulated PBMCs and AD/ADRD related plasma biomarkers. Table show the results of the logistic regression comparing PBMC T_H17 signature to the plasma biomarkers. Bolded Red font highlights statistically significant results.

Plasma biomarkers		CDR					
		both		women		men	
		FDR=	d[1]	FDR=	d[1]	FDR=	d[1]
Aβ42/Aβ40	unadjusted	0.998	0.125	0.593	0.319	0.461	0.636
	age adj	0.945		0.779		0.256	
Aβ40 (log2)	unadjusted	0.998	0.033	0.593	0.355	0.617	0.366
	age adj	0.945		0.779		0.463	
Aβ42 (log2)	unadjusted	0.998	0	0.716	0.167	0.77	0.149
	age adj	0.945		0.825		0.758	
p-tau[181] (log2)	unadjusted	0.784	0.367	0.476	0.479	0.617	0.369
	age adj	0.945		0.779		0.554	
NF-L (log2)	unadjusted	0.998	0.073	0.716	0.208	0.835	0.083
	age adj	0.945		0.825		0.846	
GFAP (log2)	unadjusted	0.998	0.208	0.016	1.104	0.617	0.348
	age adj	0.945		0.825		0.846	

Supplemental Table 14. CDR and AD/ADRD related plasma biomarkers. Table show the results of the comparing CDR to the plasma biomarkers. [1]Cohen's d. Bolded Red font highlights statistically significant results.

			unadjusted		model 1		model 2	
			FDR=	d[1]	FDR=	d[1]	FDR=	d[1]
Plasma biomarker	IL-1β	♀	0.795	0.3626	0.645	0.6859	0.917	0.7462
		♂	0.837	0.2732	0.973	1.0767	0.885	1.0792
	IL-6	♀	0.795	0.1334	0.645	0.4842	0.917	0.3922
		♂	0.406	1.6794	0.973	0.2498	0.813	1.7246
	TNFα	♀	0.752	0.4938	0.645	0.3989	0.917	0.4183
		♂	0.837	0.748	0.423	1.6877	0.885	0.243
	IL-17A	♀	0.795	2.1013	0.645	2.2173	0.917	2.6454
		♂	0.837	0.2938	0.984	0.8398	0.946	0.8115
	IL-21	♀	0.752	1.9947	0.645	1.6773	0.917	2.0524
		♂	0.837	1.5616	0.973	3.036	0.885	3.0533
	IL-22	♀	0.752	1.0126	0.645	1.4098	0.917	1.4525
		♂	0.941	0.1051	0.984	0.0293	0.968	0.0575
	CCL-20	♀	0.795	0.0884	0.879	0.0565	0.917	0.0721
		♂	0.837	2.3308	0.984	0.1369	0.946	0.1243
	Plasma T_H17	♀	0.123	0.44280	0.186	0.40279	0.194	0.42394
		♂	0.597	0.17218	0.610	0.16644	0.766	0.18327

Supplemental Table 15. Association of CDR and plasma cytokine levels. Model 1 is adjusted for age. Model 2 is adjusted for age and 0-1 vs. 2-3 cardiovascular disease (CVD) risk factors (obesity, hypertension, and hypercholesterolemia). Plasma TH17 signature is the mean z-score of IL-17A, IL-21, IL-22, and CCL20. [1]Cohen's d.

			unadjusted		model 1		model 2	
			FDR=	d[1]	FDR=	d[1]	FDR=	d[1]
PBMC T cell populations	CD3+	♀	0.148	0.329	0.191	0.457	0.175	0.334
		♂	0.086	0.081	0.916	0.035	0.919	0.034
	CD3+CD4+	♀	0.028	0.724	0.030	1.09	0.032	0.782
		♂	0.648	0.357	0.620	0.398	0.627	0.389
	CD3+CD8+	♀	0.532	0.039	0.584	0.052	0.600	0.036
		♂	0.325	0.069	0.327	0.075	0.335	0.073
	CD4+/CD8+	♀	0.028	0.188	0.30	0.280	0.032	0.202
		♂	0.806	0.046	0.899	0.053	0.909	0.051

Supplemental Table 16. Association of T cell populations in unstimulated PBMCs and T_H17 signature and plasma cytokine levels. Model 1 is a linear regression adjusted for age. Model 2 is a linear regression adjusted for age and 0-1 vs. 2-3 cardiovascular disease (CVD) risk factors (obesity, hypertension, and hypercholesterolemia). Benjamini-Hochberg false discover rate (FDR) adjusted p values. Plasma TH17 signature is the mean z-score of IL-17A, IL-21, IL-22, and CCL20. [1]Cohen's d.
